# Burden of low birth weight and short gestation from 1990–2021 and projection to 2050: assessment against 2030 malnutrition reduction targets

**DOI:** 10.3389/fped.2025.1545857

**Published:** 2025-06-24

**Authors:** Bengui Jiang, Kelly Lin, Nicholas Buys, Yanfei Qi, Jing Sun

**Affiliations:** ^1^ Department of Gynecology, Ningbo Women and Children’s Hospital, Ningbo, China; ^2^ Ningbo Gynecological Disease Clinical Medicine Research Center, Ningbo, China; ^3^ Rural Health Research Institute, Charles Sturt University, Orange, NSW, Australia; ^4^ School of Medicine and Dentistry, Griffith University, Gold Coast, QLD, Australia; ^5^ School of Health Science and Social Work, Griffith University, Gold Coast, QLD, Australia; ^6^ Centenary Institute, The University of Sydney, Sydney, NSW, Australia

**Keywords:** low birth weight, short gestation, short gestation length, preterm (birth), malnutrition

## Abstract

**Background:**

Low birth weight (LBW) and short gestation (LBWSG) are defined as birth at a gestational age <38 weeks and at a lower birth weight than the lowest risk weight of 3,500 g. Prematurity and LBW are the leading causes of global under-5 mortalities. This study aims to provide a systematic analysis of the changes in morality and disease burden of LBW and short gestation in the past 30 years to help inform health policy formation and guide resource allocation.

**Methods:**

This study utilized data from the 2021 Global Burden of Disease Study. We measured LBWSG mortality, DALYs, and YLDs in children under 20 years of age. Data from children under 20 years of age were accessed. To assess the progress in achieving the 2030 Sustainable Development Goals of a 30% reduction in LBWSG, average annual percentage change (AAPC) was calculated on a global, regional, and national level. A mixed-effects model with sodiodemographic index (SDI) and time as the main covariates was further used to forecast LBWSG mortality and DALYs from 2022 to 2050. Countries were further classified based on their SDI scores to compare AAPC and forecasted LBWSG mortality and disease burden results.

**Results:**

Countries with high-middle SDI scores showed the most significant reduction in LBWSG under-5 mortalities (AAPC = −0.84, 95% CI = −0.86 to −0.82), followed by countries with middle, high, and low-middle SDI. However, no significant improvements were identified in low SDI countries. Furthermore, the LBWSG burden worsened among 73 countries of all SDI and income levels. Forecasting results indicated an increase in LBWSG DALYs rate in children aged 5–19 years of age across low, low-middle, and high SDI countries.

**Conclusion:**

There are significant inequalities in the accessibility and quality of maternal and child healthcare between countries with high and low SDI scores. Temporal trends and forecasting results indicated increased disease burden for LBW and short gestation suggesting that 73 countries will not be able to achieve the 2030 nutritional target of a 30% reduction in preterm birth. Comprehensive policies to address maternal risk factors are required to reduce preterm birth, while upscaling of cost-effective life-saving interventions is needed to reduce the death of preterm infants.

## Introduction

Low birth weight (LBW) and short gestation (LBWSG) is defined as birth at a gestational age less than the lowest risk age of 38 weeks and a lower birth weight than the lowest risk weight of 3,500 g (1, 2). Low birth weight is a preventable public health problem associated with increased risks of premature mortality and developmental delays (3, 4). Globally, over 90% of LBW infants are born in low- to middle-income countries (LMIC), highlighting the underlying inequalities in child and maternal healthcare access and nutrition as compared with high-income countries (1, 5). As many child mortalities and morbidities in LMICs can be prevented by targeting risk factors associated with LBW, achieving a 30% reduction in LBW has been identified as one of the six global nutritional targets by WHO (6).

Similarly, short gestation or preterm infants born before 38 weeks are also at significantly greater risks of premature mortality and morbidity (7). In 2021, preterm birth accounted for 18.1% of deaths under 5 years of age, acting as the leading cause of under-5 mortalities (8). Inequalities in quality and accessibility of healthcare drive significant differences in chances of survival between preterm infants born in LMICs and high-income countries. While >90% of extremely preterm babies born in low-income countries die within the first few days following birth, only <10% of extremely preterm infants die in high-income settings (9).

The significant differences between LBW and short gestation mortality risks between LMICs and high-income countries have been explained by the poor accessibility and quality of healthcare, as well as heightened malnutrition in LMICs (10). To ensure the infant is carried until term and is born with a healthy birth weight, mothers must receive adequate maternal nutrition and quality prenatal healthcare (11). Adequate prenatal care is critical to identify and provide timely treatments for maternal comorbidities during pregnancy (10, 11). This ensures infant growth is not restricted *in utero* and that the infant is carried until term, avoiding short gestation (11). Following birth, postnatal care acts as a continuum of care for the mother and the newborn. Preterm and LBW infants with increased risk for mortality and morbidity require additional attention and further care (4). Mothers also need to be educated on how to take care of preterm and LBW infants with additional needs and risks. However, mothers and infants from LMICs are often restricted by geographic and financial barriers, preventing them from receiving the prenatal and postnatal care required (10, 12).

While timely interventions may avoid neonatal mortalities associated with preterm birth, survivors of preterm birth often still experience significant long-term impairments and disabilities ranging from developmental difficulties and compromised immune systems to cerebral palsy and chronic lung disease (13, 14). Furthermore, preterm children who survive past 5 years of age are also at risk of other chronic diseases later in life including diabetes and cardiovascular disease due to the immature development of organ systems that have interfered *in utero* due to prematurity (13). Thus, disabilities and long-term health impacts associated with preterm birth pose significant financial stress on families and national healthcare systems.

Previous studies have focused on estimating global LBWSG-associated mortality among children under 5 years of age (9). However, the burden of LBW and short gestation in terms of disability-adjusted life year (DALYs) and years loss to disability (YLDs) have not yet been evaluated on a global scale over an extensive period of time. While past studies have shown improvements in under-5 mortalities associated with LBWSG, the progress toward the 2030 nutrition target of a 30% reduction in preterm birth requires a more in-depth global analysis (15). Systematic analyses of the burden of LBWSG in addition to mortality in the past 30 years were conducted in this study to help inform health policy formation and guide resource allocation. Comparisons on the burden of LBW and short gestation across countries of different sociodemographic index (SDI) scores can provide valuable insight as significant inequalities in LBW and short gestation prevalence and mortality have been demonstrated across LMICs and high-income countries (9). Measures of SDI are a more relevant and sophisticated metric for analyzing health outcomes and disparities as they account for sociodemographic factors including education and fertility. Education has been highly correlated with health outcomes independent of income, with higher maternal education being associated with better access to prenatal care and child-rearing practices (10).

Thus, this study aimed to identify trends in LBW and short gestation mortality, DALYs, and YLDs in children under 20 years of age from 1990 to 2021. The burden of LBW and short gestation was evaluated across different age groups on a global, regional, and national level. Furthermore, differences in changes in LBW and short gestation mortality, DALYs, and YLDs were also compared across countries with different SDI scores to identify vulnerable groups that need to be targeted to reach global nutritional targets and Sustainable Development Goals (SDGs) by 2030.

## Method

### Data source

This study utilized data from the 2021 GBD study that modeled non-fatal disease burden using DisMod-MR version 2.1. This study follows the Guidelines for Accurate and Transparent Health Estimates Report (GATHER) statement and Global Burden of Disease (GBD) protocols. The GBD study is composed of a global network of collaborators aimed to promote evidence-based intervention and monitor progress toward national and international health targets, from vital registration systems, verbal autopsies, censuses, household surveys, disease-specific registers, health service contact data, and other sources. LBW is defined as birth weight under 3,500 g, while short gestation is defined as gestational periods under 38 weeks (16).

This study utilized data on disease burden as measured by disability-adjusted life years (DALYs) and years lived with disability (YLDs) and mortality using age-standardized mortality rates (ASMRs). Only data of children under 20 years of age from 1990 to 2021 were included for analysis. Data from 204 countries and territories were accessed. These countries and territories were further classified into 5 regions based on sociodemographic index (SDI) and 21 GBD regions according to geographical contiguity. This study focused on analyzing LBW and short gestation mortality and the burden of disease or morbidity. The burden of disease was measured using YLD results that only examined years of healthy life lost due to disability, meaning only morbidity is considered.

### SDI

The sociodemographic index (SDI) is a measure that is used to represent a country's social and economic development as it includes the economy as measured by lag distributed income (LDI) per capita, mean education for those 15 years and older, and total under-25 fertility rates of nations (17). This index has been used in Global Burden of Disease studies as outcomes measured by SDI correlate strongly with health outcomes. Based on the country's SDI score, each country has been classified into one of the five categories—high, high-middle, middle, low-middle, and low. Low birth weight and short gestation DALYs, YLDs, and mortality were compared across all five SDI categories across all age groups.

### Age groups

Data from children under 20 years of age were accessed in this study. Children were further divided into five age groups, including under 5, 5–9 years, 10–14 years, 15–19 years, and total under 20. Age-standardized results were also reported for the rate of DALYs, death, and YLDs.

### Statistical analysis

This study measured LBW and short gestation mortality DALYs and YLDs in children under 20 years of age. To analyze the temporal trend of LBW and short gestation mortality and disease burden in children of different age groups, the average annual percentage change (AAPC) was calculated. The measurement AAPC is a well-established summary measure used to determine epidemiological trends in child mortality and morbidity (3, 18). An estimated AAPC is based on segmented analysis over the entire data series, acting as a summary measure of a trend that transitions over a period of time (19). This is suitable to summarize and compare rates of change that are not constant over a given time period, accounting for differences in specific time points where the outcome measured (LBWSG) may differ and be affected by factors such as war and famine. In this study, changes from 1990 to 2021 were determined (19).

To calculate AAPC, LBW and short gestation DALYs, YLDs, and mortality number and rate per 100,000 persons of each age group were extracted from the GBD database. Age-standardized rates per 100,000 persons were also extracted for AAPC calculation. The AAPC was used to show a temporal trend over a 30-year period, from 1990 to 2021. While positive AAPC indicates an increasing trend, negative AAPC indicates an improvement and decreasing trend in disease burden and mortality. Statistical significance was measured using a 95% uncertainty interval (CI). Low birth weight and short gestation mortality, DALYs, and YLDs were measured for all age groups across all levels of SDI, 21 regions and 204 countries. By comparing AAPCs on multiple levels, the progress in achieving the 2030 SDG of a 30% reduction in LBW and preterm births was assessed.

## Results

### Global

**Figure 1:**
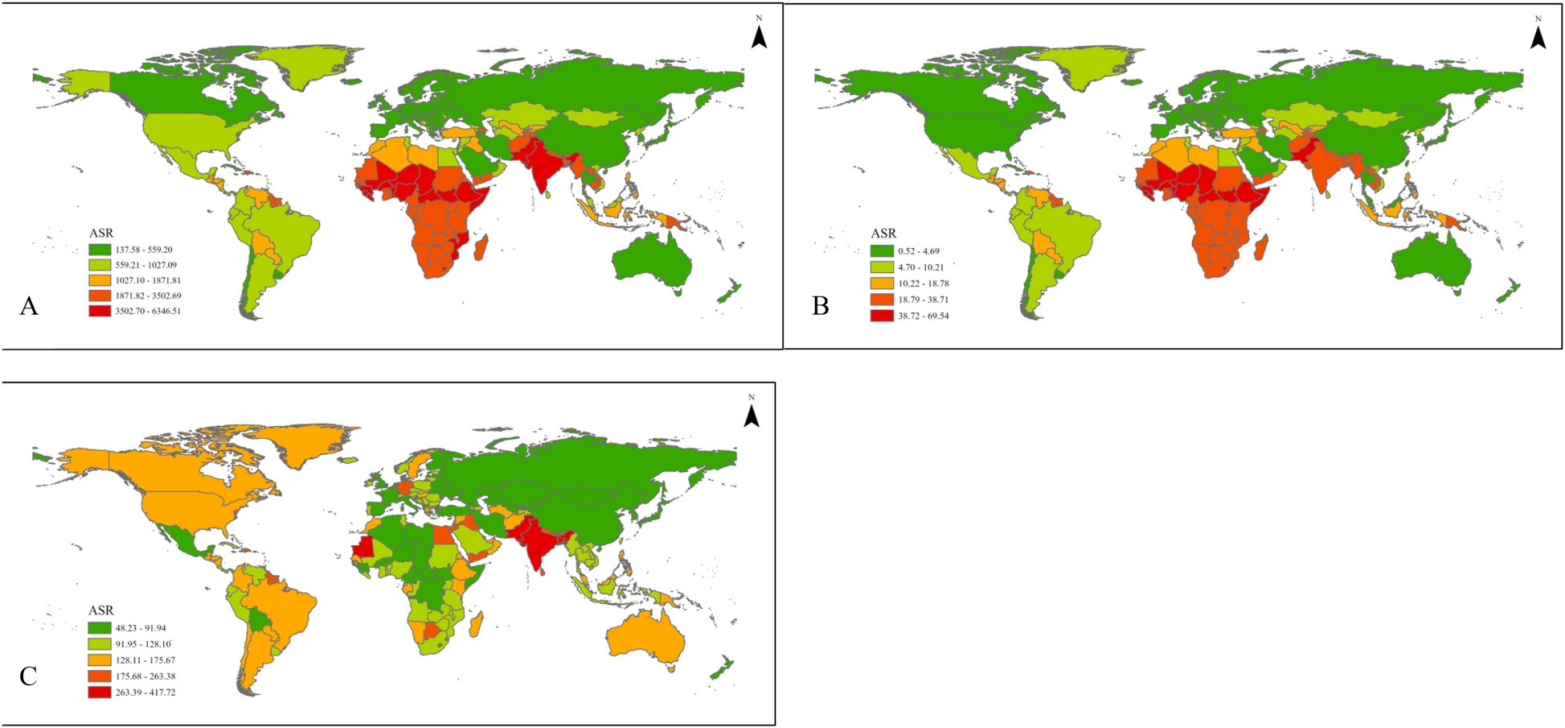
Global age-standardized results for LBW and short gestation **(A)** DALYs, **(B)** deaths, and **(C)** YLDs.

Changes in global LBWSG DALYs, YLDs, and mortality from 1990 to 2021 are presented in Table 1 and Figure 1. The number of global under-5 LBWSG deaths decreased from 3.16 million to 1.63 million, while the rate has halved from 501.11 per 100,000 to 247.07 per 100,000, with a significant AAPC of −0.47 (95% CI = −0.55 to −0.38). In contrast, LBWSG disease burden as measured by YLDs has increased globally across all age groups. In 1990, the global number of under-20 LBWSG YLDs was 2.26 million, with a rate of 122.41 per 100,000. In 2021, the number of under-20 LBWSG YLDs globally increased by 2.83 times to 6.38 million, while the rate also increased to 242.02 per 100,000, with a significant AAPC of 0.38 (95% CI = 0.28–0.48) and 0.19 (95% CI = 0.1–0.27) for the number and rate of LBWSG YLDs, respectively. For DALYs, a significant increase in children aged 5–19 years was identified. This has been further reflected in the forecasting result presented in Table 1 and Supplementary Figure S1. Forecasting results indicated an increase in DALY rate among children 5–19 years of age from 2022 to 2050. For children aged 5–9 years, the rate of LBWSG DALYs has been forecasted to increase from 237.38 to 274.76 in 2050 (95% CI = 122.48–627.61). For children aged 10–14 years, the rate of LBWSG DALYs has been forecasted to increase from 236.67 to 288.62 in 2050. For children aged between 15 and 19 years, the rate of LBWSG DALYs has been forecasted to increase from 228.60 to 311.60 in 2050.

**Table 1:** Short gestation and low birth weight DALYs, mortalities, and YLDs across countries with different SDI scores.

SDI index	Age range	1990	2021	AAPC		1990	2021	AAPC
		Rate	Rate		2050 forecasted rate	Number	Number	
Disability-adjusted life years (DALYs)								
Global	<5 years	44,673.44	22,487.95	−0.47 (−0.54, −0.38)	6,253.20 (−18,019.45, 30,525.85)	282,387,994.81	148,009,314.39	−0.43 (−0.52, −0.34)
Global	5–9 years	118.08	237.38	0.17 (0.08, 0.25)	274.76 (122.48, 627.61)	690,978.04	1,630,926.76	0.37 (0.27, 0.48)
Global	10–14 years	109.58	236.67	0.29 (0.19, 0.38)	288.62 (119.95, 707.53)	588,113.97	1,577,724.43	0.6 (0.48, 0.72)
Global	15–19 years	99.62	228.6	0.44 (0.33, 0.55)	311.60 (104.83, 944.04)	517,643.77	1,426,445.85	0.73 (0.6, 0.86)
Global	<20 years	12,499.49	5,791.11	−0.51 (−0.58, −0.43)	−1,601.99 (−7,707.84, 4,503.10)	284,184,730.59	152,644,411.43	−0.42 (−0.5, −0.33)
Global	Age-standardized	11,049.75	5,994.085	–		–	–	–
High SDI	<5 years	7,164.05	2,858.65	−0.59 (−0.63, −0.56)	−877.84 (0, 4,800.15)	4,129,497.41	1,539,273.71	−0.64 (−0.68, −0.61)
High SDI	5–9 years	148.58	147.68	−0.02 (−0.11, 0.06)	168.98 (117.40, 220.55)	85,989.97	86,694.66	−0.08 (−0.16, −0.01)
High SDI	10–14 years	142.79	145.47	0 (−0.08, 0.09)	146.04 (93.56, 198.52)	81,618.32	87,258.78	−0.03 (−0.1, 0.06)
High SDI	15–19 years	134.67	141.68	0.03 (−0.05,0.11)	145.04 (127.12, 166.14)	82,804.49	85,268.99	−0.06 (−0.13, 0.02)
High SDI	<20 years	1,870.45	772.81	−0.58 (−0.61, −0.54)	498.72 (0, 1,002.01)	4,379,910.19	1,798,496.13	−0.61 (−0.64, −0.58)
High SDI	Age-standardized	1,921.31	847.37	–		–	–	–
High-middle SDI	<5 years	19,433.88	3,513.09	−0.78 (−0.81, −0.75)	−6,953.59 (−14,501.27, 594.08)	20,309,598.7	2,460,738.55	−0.84 (−0.86, −0.81)
High-middle SDI	5–9 years	123.46	126.12	−0.05 (−0.16, 0.06)	103.08 (23.58, 182.57)	124,489.3	103,936.55	−0.14 (−0.24, −0.04)
High-middle SDI	10–14 years	118.39	127.17	0 (−0.11, 0.11)	132.53 (72.90, 244.43)	116,261.06	99,746.65	−0.12 (−0.23, −0.03)
High-middle SDI	15–19 years	108.41	126.52	0.11 (−0.02, 0.24)	127.33 (72.66, 226.27)	110,265.49	91,692.5	−0.17 (−0.26, −0.07)
High-middle SDI	<20 years	5,098.24	908.52	−0.78 (−0.81, −0.75)	−2,173.36 (−5,856.22, 1,509.50)	20,660,614.55	2,756,114.25	−0.82 (−0.84, −0.8)
High-middle SDI	Age-standardized	4,265.50	1,003.11	–		–	–	–
Middle SDI	<5 years	32,119.56	11,185.41	−0.62 (−0.68, −0.56)	−8,815.26 (−17,858.26, 227.74)	65,791,305.28	19,755,352.38	−0.67 (−0.72, −0.62)
Middle SDI	5–9 years	110.93	204.56	0.22 (0.11, 0.32)	204.70 (128.52, 280.87)	215,130.4	403,122.82	0.24 (0.13, 0.35)
Middle SDI	10–14 years	102.58	205.73	0.33 (0.21, 0.44)	276.37 (107.68, 722.33)	187,309.72	397,415.15	0.4 (0.28, 0.52)
Middle SDI	15–19 years	91.13	204.86	0.53 (0.39, 0.66)	260.14 (0, 520.65)	169,102.06	373,530.63	0.49 (0.36, 0.62)
Middle SDI	<20 years	8,653.11	2,793.6	−0.65 (−0.7, −0.59)	−3,317.47 (−9,802.29, 3,167.36)	66,362,847.47	20,929,420.98	−0.65 (−0.7, −0.6)
Middle SDI	Age-standardized	7,804.172	3,047.03	–		–	–	–
Low-middle SDI	<5 years	70,123.67	30,531.3	−0.54 (−0.61, −0.45)	−3,636.88 (0, −2,775.55)	117,951,943.92	58,491,214.75	−0.49 (−0.57, −0.4)
Low-middle SDI	5–9 years	131.44	359.81	0.14 (0.04, 0.26)	409.64 (229.96, 737.71)	200,407.03	701,155.39	0.39 (0.27, 0.54)
Low-middle SDI	10–14 years	116.36	354.21	0.25 0.13, 0.37)	372.44 (236.41, 508.48)	154,434.48	685,030.84	0.75 (0.58, 0.92)
Low-middle SDI	15–19 years	102.39	336.52	0.35 (0.22, 0.49)	404.74 (18.13, 791.34)	119,991.46	621,041.77	1.09 (0.89, 1.31)
Low-middle SDI	<20 years	20,755.1	7,914.63	−0.6 (−0.65, −0.52)	−3,384.76 (0, −520.16)	118,426,776.89	60,498,442.75	−0.48 (−0.55, −0.38)
Low-middle SDI	Age-standardized	17,297.07	8,161.95	–		–	–	–
Low SDI	<5 years	76,707.34	39,664.05	−0.47 (−0.56, −0.37)	1,545.05 (−24,722.78, 27,812.89)	74,080,364.23	65,673,867.75	−0.03 (−0.19, 0.15)
Low SDI	5–9 years	80.92	218.3	0.16 (0.04, 0.31)	317.85 (103.77, 991.90)	64,514.9	335,083.29	1.35 (1.1, 1.65)
Low SDI	10–14 years	73.18	217.78	0.28 (0.15, 0.44)	329.54 (92.82, 1,192.01)	48,090.63	307,405.97	1.9 (1.6, 2.25)
Low SDI	15–19 years	65.82	204.99	0.38 (0.23, 0.56)	292.90 (0, 614.40)	35,118.42	254,142.38	2.38 (2, 2.83)
Low SDI	<20 years	25,130.48	11,395.04	−0.53 (−0.61, −0.45)	−1,859.64 (−8,161.52, 4,442.25)	74,228,088.19	66,570,499.39	−0.02 (−0.18, 0.16)
Low SDI	Age-standardized	19,469.43	10,424.48	–		–	–	–
Deaths								
Global	<5 years	501.11	247.07	−0.47 (−0.55, −0.38)	605.81 (431.21, 780.41)	3,167,605.32	1,626,132.54	−0.44 (−0.52, −0.34)
Global	<20 years	139.32	61.69	−0.52 (−0.59, −0.44)	468.77 (356.64, 579.38)	3,167,605.32	1,626,132.54	−0.44 (−0.52, −0.34)
Global	Age-standardized	120.60	63.95	–		–	–	–
High SDI	<5 years	78.95	30.14	−0.6 (−0.64, −0.57)	193.73 (0.00, 442.63)	45,509.1	16,228.85	−0.65 (−0.69, −0.62)
High SDI	<20 years	19.43	6.97	−0.63 (−0.66, −0.59)	187.38 (130.32, 244.44)	45,509.1	16,228.85	−0.65 (−0.69, −0.62)
High SDI	Age-standardized	19.73	7.80	–		–	–	–
High-middle SDI	<5 years	217.28	37.64	−0.79 (−0.82, −0.76)	142.39 (108.15, 176.16)	227,072.77	26,367.39	−0.84 (−0.86, −0.82)
High-middle SDI	<20 years	56.03	8.69	−0.81 (−0.83, −0.78)	72.58 (54.42, 90.74)	227,072.77	26,367.39	−0.84 (−0.86, −0.82)
High-middle SDI	Age-standardized	45.99	9.74	–		–	–	–
Middle SDI	<5 years	360.06	121.95	−0.63 (−0.69, −0.57)	42.75 (33.08, 52.29)	737,513.01	215,377.55	−0.67 (−0.72, −0.62)
Middle SDI	<20 years	96.16	28.75	−0.67 (−0.72, −0.61)	−2.40	737,513.01	215,377.55	−0.67 (−0.72, −0.62)
Middle SDI	Age-standardized	84.95	31.56	–		–	–	–
Low-middle SDI	<5 years	787.12	334.98	−0.54 (−0.61, −0.46)	41.32 (33.03, 49.61)	1,323,975.28	641,750.55	−0.49 (−0.57, −0.4)
Low-middle SDI	<20 years	232.04	83.96	−0.61 (−0.67, −0.54)	41.52 (30.73, 52.18)	1,323,975.28	641,750.55	−0.49 (−0.57, −0.4)
Low-middle SDI	Age-standardized	188.73	86.70	–		–	–	–
Low SDI	<5 years	861.64	438.13	−0.47 (−0.56, −0.37)	203.11 (152.76, 252.78)	832,131.3	725,431.63	−0.03 (−0.19, 0.15)
Low SDI	<20 years	281.72	124.17	−0.54 (−0.61, −0.45)	143.29 (108.53, 178.05)	832,131.3	725,431.63	−0.03 (−0.19, 0.15)
Low SDI	Age-standardized	214.28	113.40	–		–	–	–
Years lived with disability (YLDs)								
Global	<5 years	156.04	264.98	0.02 (−0.06, 0.09)		2,001,631.68	1,744,051.9	0.08 (0, 0.15)
Global	5–9 years	118.08	237.38	0.17 (0.08, 0.25)		149,357.22	1,630,926.76	0.37 (0.27, 0.48)
Global	10–14 years	109.58	236.67	0.29 (0.19, 0.38)		105,312.57	1,577,724.43	0.6 (0.48, 0.72)
Global	15–19 years	99.62	228.6	0.44 (0.33, 0.55)		106,428.77	1,426,445.85	0.73 (0.6, 0.86)
Global	<20 years	122.41	242.02	0.19 (0.1, 0.27)		2,362,730.24	6,379,148.94	0.38 (0.28, 0.48)
Global	Age-standardized	202.52	242.26	–		–	–	–
High SDI	<5 years	153.75	147.89	−0.04 (−0.11, 0.03)		6,919.26	79,634.83	−0.16 (−0.23, −0.1)
High SDI	5–9 years	148.58	147.68	−0.02 (−0.11, 0.06)		13,107.68	86,694.66	−0.08 (−0.16, −0.01)
High SDI	10–14 years	142.79	145.47	0 (−0.08, 0.09)		11,407.48	87,258.78	−0.03 (−0.1, 0.06)
High SDI	15–19 years	134.67	141.68	0.03 (−0.05, 0.11)		13,216.24	85,268.99	−0.06 (−0.13, 0.02)
High SDI	<20 years	144.79	145.61	−0.01 (−0.08, 0.07)		44,650.66	338,857.26	−0.08 (−0.15, −0.01)
High SDI	Age-standardized	147.26	145.76	–		–	–	–
High-middle SDI	<5 years	135.16	127.29	−0.12 (−0.22, −0.02)		126,049.91	89,159.50	−0.33 (−0.41, −0.26)
High-middle SDI	5–9 years	123.46	126.12	−0.05 (−0.16, 0.06)		22,316.07	103,936.55	−0.14 (−0.24, −0.04)
High-middle SDI	10–14 years	118.39	127.17	0 (−0.11, 0.11)		17,708.95	99,746.65	−0.12 (−0.23, −0.03)
High-middle SDI	15–19 years	108.41	126.52	0.11 (−0.02, 0.24)		19,403.99	91,692.5	−0.17 (−0.26, −0.07)
High-middle SDI	<20 years	121.47	126.76	−0.02 (−0.13, 0.08)		185,478.92	384,535.2	−0.19 (−0.29, −0.11)
High-middle SDI	Age-standardized	129.46	126.78	–		–	–	–
Middle SDI	<5 years	137.08	215.57	0.1 (0, 0.19)		457,609.46	380,734.07	−0.03 (−0.12, 0.04)
Middle SDI	5–9 years	110.93	204.56	0.22 (0.11, 0.32)		42,164.61	403,122.82	0.24 (0.13, 0.35)
Middle SDI	10–14 years	102.58	205.73	0.33 (0.21, 0.44)		32,875.8	397,415.15	0.4 (0.28, 0.52)
Middle SDI	15–19 years	91.13	204.86	0.53 (0.39, 0.66)		35,285.69	373,530.63	0.49 (0.36, 0.62)
Middle SDI	<20 years	111.13	207.53	0.27 (0.16, 0.37)		567,935.56	1,554,802.67	0.24 (0.13, 0.34)
Middle SDI	Age-standardized	163.82	207.78	–		–	–	–
Low-middle SDI	<5 years	193.26	396.19	−0.06 (−0.13, 0.02)		919,238.61	759,014.81	0.04 (−0.04, 0.12)
Low-middle SDI	5–9 years	131.44	359.81	0.14 (0.04, 0.26)		49,776.66	701,155.39	0.39 (0.27, 0.54)
Low-middle SDI	10–14 years	116.36	354.21	0.25 (0.13, 0.37)		30,655.8	685,030.84	0.75 (0.58, 0.92)
Low-middle SDI	15–19 years	102.39	336.52	0.35 (0.22, 0.49)		27,625.39	621,041.77	1.09 (0.89, 1.31)
Low-middle SDI	<20 years	140.19	361.89	0.11 (0.02, 0.21)		1,027,296.46	2,766,242.81	0.44 (0.32, 0.56)
Low-middle SDI	Age-standardized	318.74	362.28	–		–	–	–
Low SDI	<5 years	155.36	262.41	−0.08 (−0.16, 0)		491,077.48	434,491.99	0.67 (0.53, 0.82)
Low SDI	5–9 years	80.92	218.3	0.16 (0.04, 0.31)		21,934.05	335,083.29	1.35 (1.1, 1.65)
Low SDI	10–14 years	73.18	217.78	0.28 (0.15, 0.44)		12,618.91	307,405.97	1.9 (1.6, 2.25)
Low SDI	15–19 years	65.82	204.99	0.38 (0.23, 0.56)		10,847.12	254,142.38	2.38 (2, 2.83)
Low SDI	<20 years	100.81	227.85	0.09 (−0.01, 0.2)		536,477.55	1,331,123.64	1.28 (1.08, 1.5)
Low SDI	Age-standardized	199.20	226.41	–		–	–	–

**Figure 2:**
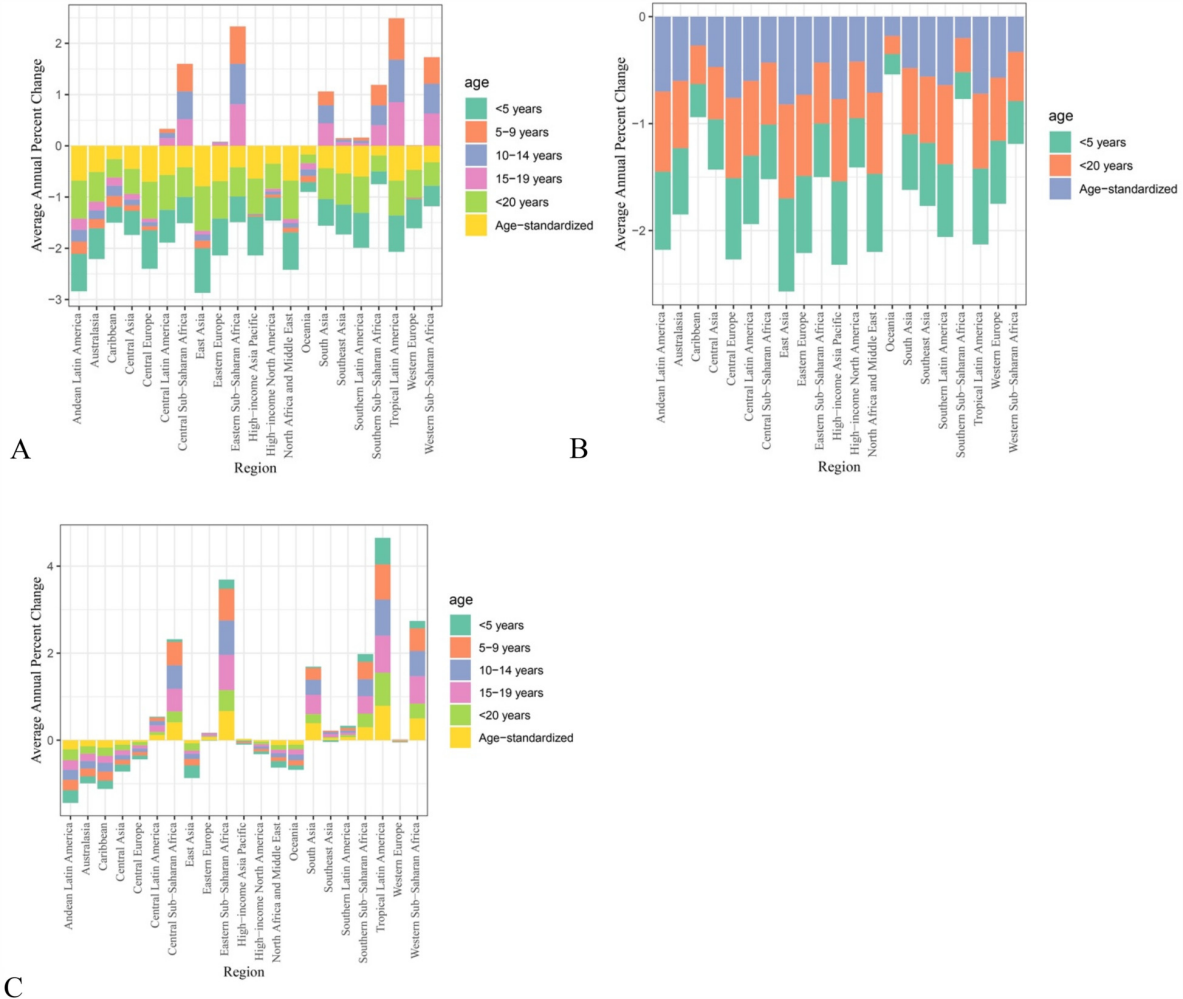
AAPC for LBW and short gestation **(A)** DALYs, **(B)** deaths, and **(C)** YLDs, classified based on geographical regions.

### Sociodemographic index

When countries are grouped based on their SDI scores, the greatest improvement in LBW and short gestation mortality, DALYs, and YLDs is observed in the high-middle SDI group, followed by the high or middle SDI index group across all age groups. In contrast, although the number of under-5 mortalities due to LBWSG decreased from 832,131.3 to 725,431.63 in low SDI countries, the AAPC indicated no significant improvements (AAPC = −0.03, 95% CI = −0.19 to 0.15).

In terms of LBW and short gestation YLDs, significant improvements were observed in under-5 children in high-middle SDI (AAPC = −0.33, 95% CI = −0.41 to −0.26) and high SDI (AAPC = −0.16, 95% CI = −0.23 to −0.1) countries only. No significant improvements were observed in middle (AAPC = −0.03, 95% CI = −0.12 to 0.04) and low-middle SDI (AAPC = 0.04, 95% CI = −0.04 to 0.12) countries from 1990 to 2021. Notably, significant increases in under-5 LBW and short gestation YLDs were found in countries with low SDI scores. From 1990 to 2021, the number of under-20 LBWSG YLDs increased by almost 100% from 536,477.55 to 1,331,123.64, with a positive AAPC of 0.67 (95% CI = 0.53–0.82). Across age groups, LBW and short gestation burden of disease increased for children aged 5–9, 10–14, and 15–19 years in middle, low-middle, and low SDI countries. Overall, LBW and short gestation YLDs only improved across all age groups from 0 to 19 years of age in high-middle SDI countries. As shown in Table 1 and Supplementary Figure S1, disease burden as measured in LBW and short gestation DALYs have also been forecasted to increase from 2022 to 2050 in children aged 5–9 years of age in high (forecasted rate = 168.98, 95% CI = 117.40–220.55), low-middle (forecasted rate = 409.64, 95% CI = 229.96–737.71), and low SDI countries (forecasted rate = 317.85, 95% CI = 103.77–991.90).

### Regional

Countries included for analyses were divided into different regions based on their geographical locations. Regional results have been displayed in Supplementary Table 1 and Figure 2.

In terms of LBW and short gestation DALYs and YLDs, some regions showed significant improvements. Under-5 LBW and short gestation DALYs and YLDs improved in Central Asia (AAPC = −0.44, 95% CI = −0.53 to −0.34), Central Europe (AAPC = −0.45, 95% CI = −0.5 to −0.39), East Asia (AAPC = −0.46, 95% CI = −0.54 to −0.36), high-income Asia Pacific (AAPC = −0.19, 95% CI = −0.33 to −0.02), Oceania (AAPC = −0.49, 95% CI = −0.54 to −0.44), South Asia (AAPC = −0.15, 95% CI = −0.28 to −0.03), and Western Europe (AAPC = −0.4, 95% CI = −0.49 to −0.29). In contrast, some regions showed significant increases in LBW and short gestation YLDs in children under 5 years of age, with positive AAPCs. This included Australasia (AAPC = 0.54, 95% CI = 0.18–0.98), Eastern Europe (AAPC = 0.85, 95% CI = 0.6–0.11), and Southeast Asia (AAPC = 0.73, 95% CI = 0.49–1.01).

Similarly, contrasting results of LBW and short gestation YLDs were found in older age groups. Most regions showed no significant improvement across children aged 5–9, 10–14, and 15–19 years. However, some countries showed significant improvements such as Eastern Europe (AAPC = −0.68, 95% CI = −0.74 to −0.61), Central Asia (AAPC = −0.6, 95% CI = −0.67 to −0.53), and East Asia (AAPC = −0.25, 95% CI = −0.38 to −0.09).

### National

As presented in Supplementary Table S2, varying national trends in LBW and short gestation-associated morbidities and disabilities were found.

These results also varied within countries of the same SDI level. The disease burden of under-20 LBWSG as measured by YLDs increased in a total of 73 countries, including countries with low to high SDI scores.

Overall, the most significant increase in age-standardized rate of LBWSG YLDs were found in many low to middle SDI countries in the sub-Saharan African region including Angola, which increased from 98.14 to 144.86, Ethiopia from 71.16 to 193.71, Cameroon from 80.52 to 96.91, Central African Republic from 88.68 to 99.08, Gambia from 279.75 to 242.13, Congo from 152.84 to 195.73, Democratic Republic of the Congo from 78.95 to 101.67, Ghana from 140.91 to 147.14, Madagascar from 155.42 to 210.40, Guinea from 85.64 to 111.04, Liberia from 115.81 to 177.35, Sao Tome and Principe from 188.69 to 242.70, Senegal from 158.73 to 222.37, Uganda from 121.08 to 144.21, Chad from 61.17 to 72.02, United Republic of Tanzania from 132.46 to 171.12, Zambia from 121.22 to 165.88, Zimbabwe from 166.44 to 173.08, Mozambique from 127.52 to 182.44, and Kenya from 109.75 to 197.76.

Beyond the sub-Saharan region, similar increases in LBWSG YLDs have been identified in some South American countries including Brazil from 111.01 to 201.14, Chile from 133.94 to 155.13, Portugal from 106.01 to 116.50, Argentina from 160.22 to 162.20, and Mexico from 91.56 to 117.55. In Asia, increased rates of LBWSG YLDs have also been identified in Indonesia from 82.08 to 124.68, the Philippines from 151.89 to 201.43, and India from 404.98 to 555.97.

Some countries with high SDI scores also showed an increase in LBWSG YLDs, accounting for the forecasted increase of LBWSG disease burden to 2050 found in high SDI countries presented in Table 1 and Supplementary Figure S1. These countries included high-income countries including Austria from 127.21 to 145.75, Finland from 83.24 to 85.40, Canada from 143.12 to 152.66, Greece from 64.19 to 155.13, Japan from 81.82 to 91.14, Malta from 125.20 to 132.29, Monaco from 113.56 to 114.87, the Netherlands from 92.07 to 103.94, Belgium from 82.74 to 100.32, Poland from 116.93 to 124.89, Russia from 98.88 to 103.25, and Sweden from 144.87 to 158.45.

## Discussion

Striking differences in progress toward achieving the 2030 SDG target of a 30% reduction in LBW and preterm birth were found across countries with different SDI regions (6). Countries with low SDI scores showed no significant improvements in LBWSG mortality since 1990, while countries with high to middle SDI scores showed significant improvements in LBWSG mortality. Despite the improvements in LBWSW mortalities, disease burden as measured by DALYs and YLDs has increased in countries of all SDI scores from low to high SDI, with a similar forecasted increase in LBWSG disease burden in low, low-middle, and high SDI countries by 2050. The greatest percentage increase in under-20 age-standardized LBWSG disease burden was in low SDI countries of the sub-Saharan region, including the Central African Republic, Democratic Republic of the Congo, Ethiopia, Ghana, Liberia, Senegal, Gambia, Kenya, Mozambique, and Guinea. Low to middle SDI countries in the South America region such as Argentina, Chile, and Brazil and Southeast Asia such as Indonesia, the Philippines, and India also showed an increase in LBWSG disease burden. Countries with high SDI scores across Europe including Belgium, Greece, Austria, Monaco, and Finland and high SDI Asian countries such as Japan also presented an increasing trend in LBWSG disease burden from 1990 to 2021. These increasing trends in LBWSG YLDs explain the forecasted global increase of the LBWSG disease burden to 2050, despite the significant decrease in mortality across low-middle to high SDI countries. Thus, despite global efforts to reduce preterm births and associated mortalities, results from this study indicated that 73 countries are not on track to achieve the 2030 target. This aligns with the most recent report on 2030 nutritional targets that projected no countries will reach the target of a 30% decrease in the prevalence of LBWSG (20).

In line with the current study, previous studies have identified a wide survival gap between preterm infants born in LMICs and high-income countries (9, 14). While >90% of extremely preterm babies born in low-income countries die within the first few days following birth, only <10% of extremely preterm infants die in high-income settings (9). Differences in LBW and short gestation mortality between countries with different SDI scores indicate that significant inequalities in the quality of healthcare and sociodemographic factors still exist between high, middle, and low SDI countries. Before birth, adequate prenatal care is required to reduce the risk of LBWSG. Routine screening in prenatal care for hypertensive diseases, monitoring of blood sugar, and advice on pregnancy nutrition are all critical in addressing known maternal risk factors of hypertension, diabetes, and malnutrition associated with LBWSG (13, 21, 22). Previous studies have found that only 11.3% of mothers in LMICs receive the WHO-recommended minimum of eight prenatal health checks before giving birth. In some sub-Saharan African countries such as Rwanda, Niger, Senegal, Chad, Tanzania, and Zambia, <5% of mothers receive the recommended minimum of eight prenatal health checks (23). This aligns with the current study that found the greatest disease burden of LBWSG in sub-Saharan regions.

Following birth, preterm and LBW infants are at a greater risk of poor health outcomes associated with child mortality due to life-threatening complications including severe infections, respiratory distress syndrome, brain injuries, and anemia of prematurity (14). Thus, neonates born preterm with LBW require timely postnatal care to avoid premature mortality and to ensure healthy development as complications of prematurity are the most common cause of death in children under 5 years of age (15). However, in LMICs, financial and geographical barriers significantly limit access to quality postnatal care required to avoid premature death. Lack of access to quality prenatal and postnatal care in LMICs both contribute to a greater prevalence of LBWSG disease burden and mortality.

Survivors of preterm birth often have different levels of disabilities or long-term health impacts ranging from developmental difficulties and compromised immune systems to chronic lung disease and cerebral palsy (14). Long-term health consequences and disabilities associated with preterm birth cannot be reversed and pose a significant burden on families and national healthcare systems as ongoing care is required to support long-term wellbeing (24). This has been demonstrated in the current study where disabilities associated with LBWSG as measured by YLDs have only increased across countries of all SDI levels.

The significant increase in LBW and short gestation YLDs with reduced under-5 mortality rates in low-middle and middle SDI countries indicates that while more premature and LBW infants are surviving past 5 years of age, these children are living with disabilities and morbidities associated with LBW or short gestation, contrasting with the striking improvements in mortality in middle, high-middle, and high SDI countries. Regardless of the improvement in preterm mortality, survivors of preterm birth often develop disabilities and long-term health consequences that require continuous care (25). This poses a significant financial burden on families and national healthcare systems, making such care inaccessible for many families in LMICs. Thus, in addition to aims to reduce under-5 mortality, a specific target for a 30% reduction in preterm birth by 2030 has been set by the WHO to achieve SDG 3 of good health and wellbeing (14). However, forecasting results calculated based on trends in the past 30 years indicate that disabilities associated with LBW and short gestation as measured by DALYs and YLDs are only predicted to increase in 2050 for children aged 5–19 years globally across 73 countries. Similarly, a recent report projected that no country would reach the 2030 nutritional target of a 30% decrease in the prevalence of LBWSG (20). These alarming statistics highlight the need for a comprehensive plan to address the different maternal risk factors for mothers in high-income and LMICs.

To prevent under-5 death and reduce long-term disabilities associated with preterm birth, comprehensive care to support both pregnant mothers and preterm infants is required.

Preterm infants born in low SDI countries still require access to timely life-saving treatment to reduce mortalities associated with LBWSG. Upscaling of low-cost life-saving interventions including thermal care, kangaroo mother care, feeding support, and infection control in low SDI countries may help save the lives of premature infants (14). Upscaling of low-cost interventions aims to achieve adequate coverage through improving accessibility, while ensuring cost-effectiveness to maximize the impact of national health policies in reducing mortality and morbidity in low SDI countries with minimal health budget. One low-cost intervention strongly recommended by WHO is breastfeeding either through the infant's mother's own milk or donor human milk when the mother's own milk is not available. Exclusive breastfeeding until 6 months of age is considered one of the most cost-effective interventions to improve child health and wellbeing (26). Breastmilk consists of a wide range of bioactive compounds including immunoglobulins, lactoferrin, and cytokines critical to help improve immunity, reducing risks of gastrointestinal infections, respiratory tract infections, urinary tract infections, and hospital admissions associated with increased mortality (27). Previous studies have indicated the effectiveness of exclusive breastfeeding on weight gain and growth in length and head circumference in LBWSG infants, indicating that breast milk is the most preferable choice for feeding in LBWSG infants (28). In addition to its physiological benefits on infant growth and development, previous reviews have indicated that breastfeeding promotion is associated with greater health benefits and lower costs than usual care, suggesting the promotion of breastfeeding as a cost-effective intervention (29, 30). Thus, with adequate prenatal and postnatal education in low SDI countries, optimal breastfeeding can act as a cost-effective intervention to reduce mortality associated with LBWSG.

In addition to policies aimed at reducing mortalities, policies and interventions to reduce the prevalence of LBWSG in LMICs require nations to address known maternal risk factors for premature births including chronic maternal malnutrition, poor prenatal care, and malaria and urinary tract infections (10, 21, 22). National policies and funding to help make quality prenatal care affordable and accessible for expecting mothers in LMICs are required to achieve the minimum recommended eight prenatal checks. Previous studies have shown a decreased occurrence of preterm birth with increased antenatal care visits in LMICs such as Bangladesh (31). Improving access to prenatal healthcare may help identify mothers at risk of preterm birth through regular screening of blood pressure and blood glucose levels, while education on nutrition can help address malnutrition that is common among pregnant mothers in LMICs. As over 60% of preterm births occur in LMICs, addressing maternal risk factors that contribute to LBWSG in LMICs will aid in achieving the 2030 target (5). Lastly, support for long-term disabilities associated with preterm birth is also required across all demographic levels and settings to address the increasing trend of LBWSG disabilities identified.

Despite the significant trends identified in this study, some limitations should be considered. This study categorized countries based on their SDI scores in 2021, meaning that movements of countries between SDI categories due to economic growth, political changes, health improvements, or setbacks were not considered. Another limitation that may have influenced the AAPC scores calculated was that LBWSG mortality and disease burden may not have been well documented in 1990, especially in countries with low SDI scores with worse accessibility and quality of healthcare. Thus, the lack of significant improvement in LBWSG mortality identified in low SDI countries may be due to increased numbers recorded in 2021. Nonetheless, our results were supported by previous studies that also found no measurable change in short gestation in the last decade (9). Although LBWSG is often measured separately, the GBD study grouped LBW and short gestation together as one risk factor. While each factor may have varying levels of effects, LBW and short gestation are both linked to similar prenatal risk factors of chronic malnutrition and poor prenatal healthcare. LBWSG is also associated with an increased risk of mortality and long-term disability through similar mechanisms of increased risk of infection and poor physical and cognitive development. Thus, policies to address underlying prenatal risk factors and life-saving interventions for LBWSG infants would be similar.

## Conclusion

Although significant global reductions of LBW and short gestation under-5 mortalities have been found in the past 30 years, improvements only occurred in countries with high to middle SDI scores. The lack of improvements and the significantly higher prevalence of under-5 LBW and short gestation mortalities in low SDI countries highlight the significant inequalities in accessibility and quality of maternal and child healthcare between countries with high and low SDI scores. The forecasted increase in LBW and short gestation disabilities in high, low-middle, and low SDI countries as measured by YLDs and DALYs further suggested that many countries are not on track to achieve the 2030 SDG target of a 30% reduction in preterm births. Thus, current global targets are not only focused on reducing mortalities associated with preterm birth, but the reduction in the prevalence of preterm birth is important to address associated long-term disabilities that burden families and national healthcare systems. Achieving the 2030 SDG target will require comprehensive policies to support family planning, access to quality prenatal care, and maternal nutrition to address risk factors that contribute to preterm birth.

## Data Availability

To download GBD data used in the analyses, please visit the GBD 2021 Sources Tool website. To download the forecasted estimates used in the analyses, please visit the GBD visualization tools.
