# Supplementary material for: Burden of low birth weight and short gestation from 1990–2021 and projection to 2050: assessment against 2030 malnutrition reduction targets
Source: Front Pediatr. 2025 Jun 24;13:1545857. doi: 10.3389/fped.2025.1545857 (PMC12234553; doi:10.3389/fped.2025.1545857)
Supplement: Supplementary file 1 [file Table1.docx]

**Table 2: Short gestation and low birth weight DALYs, mortalities and YLDs in different geographical regions**

|  |  | **1990** | **2021** | **AAPC** | **1990** | **2021** | **AAPC** |
| --- | --- | --- | --- | --- | --- | --- | --- |
|  |  | Rate | Rate |  | Number | Number |  |
| **DALYs (Disability-Adjusted Life Years)** | |  |  |  |  |  |  |
| Andean Latin America | <5 years | 34475.03 | 8399.29 | -0.73 (-0.79, -0.66) | 1877713.83 | 517043.81 | -0.18 (-0.36, 0.06) |
| Andean Latin America | 5-9 years | 114.04 | 136.17 | -0.24 (-0.38, -0.04) | 5671.66 | 8295.72 | -0.17 (-0.36, 0.06) |
| Andean Latin America | 10-14 years | 107.86 | 131.45 | -0.23 (-0.38, -0.06) | 4988.12 | 7685.36 | -0.17 (-0.37, 0.09) |
| Andean Latin America | 15-19 years | 99.96 | 123.43 | -0.22 (-0.37, -0.01) | 4089.75 | 6885.88 | -0.51 (-0.57, -0.43) |
| Andean Latin America | <20 years | 9889.7 | 2280.65 | -0.7 4(-0.8, -0.68) | 1892463.35 | 539910.77 | -0.58 (-0.63, -0.5) |
| Andean Latin America | Age-standardized | 8107.64 | 2270.65 | - | - | - | - |
| Australasia | <5 years | 5828.22 | 2316.87 | -0.6 (-0.67, -0.53) | 89917.99 | 42075.54 | -0.25 (-0.39,-0.09) |
| Australasia | 5-9 years | 164.92 | 147.24 | -0.18 (-0.36, 0.06) | 2519.11 | 2878.61 | -0.2 (-0.35, -0.03) |
| Australasia | 10-14 years | 161.15 | 144.37 | -0.17 (-0.36, 0.06) | 2444.23 | 2829.72 | -0.32 (-0.44, -0.17) |
| Australasia | 15-19 years | 154.78 | 140.12 | -0.17 (-0.37, 0.09) | 2612.42 | 2537.15 | -0.4 (-0.49, -0.29) |
| Australasia | <20 years | 1553.71 | 667.23 | -0.58 (-0.63, -0.5) | 97493.76 | 50321.02 | -0.33 (-0.43, -0.21) |
| Australasia | Age-standardized | 1645.82 | 705.96 | - | - | - | - |
| Caribbean | <5 years | 32692.34 | 23638.77 | -0.31 (-0.45, -0.14) | 1353167.17 | 914390.13 | -0.87 (-0.89, -0.84) |
| Caribbean | 5-9 years | 179.17 | 234.29 | -0.21 (-0.3, -0.08) | 6675.84 | 9032.43 | -0.15 (-0.28, -0.03) |
| Caribbean | 10-14 years | 172.67 | 224.91 | -0.2 (-0.3, -0.08) | 6127.2 | 8505.11 | -0.12 (-0.25, 0.01) |
| Caribbean | 15-19 years | 160.43 | 211.17 | -0.16 (-0.26, -0.04) | 5895.42 | 7934.51 | -0.07 (-0.2, 0.07) |
| Caribbean | <20 years | 9092.24 | 6157.96 | -0.36 (-0.48, -0.21) | 1371865.63 | 939862.18 | -0.79 (-0.83, -0.75) |
| Caribbean | Age-standardized | 9058.92 | 6284.06 | - | - | - | - |
| Central Asia | <5 years | 24317.96 | 12499.4 | -0.47 (-0.55, -0.38) | 2305171.91 | 1249568.06 | -0.12 (-0.27, 0.07) |
| Central Asia | 5-9 years | 104.46 | 111.11 | -0.11 (-0.24, 0.04) | 8605.28 | 10476.61 | -0.17 (-0.32, 0) |
| Central Asia | 10-14 years | 99.65 | 106.98 | -0.11 (-0.25, 0.02) | 7213.41 | 8825.45 | -0.17 (-0.33, 0.01) |
| Central Asia | 15-19 years | 93.45 | 100.55 | -0.11 (-0.24, 0.04) | 6154.5 | 6986.78 | -0.76 (-0.79, -0.73) |
| Central Asia | <20 years | 7377.97 | 3684.82 | -0.49 (-0.57, -0.4) | 2327145.1 | 1275856.91 | -0.03 (-0.18, 0.12) |
| Central Asia | Age-standardized | 6195.76 | 3313.98 | - | - | - | - |
| Central Europe | <5 years | 12150.29 | 3005.88 | -0.75 (-0.79, -0.71) | 1085467.82 | 167898.54 | -0.73 (-0.77, -0.68) |
| Central Europe | 5-9 years | 138.9 | 131.33 | -0.08 (-0.2, 0.03) | 13424.61 | 7655.19 | -0.71 (-0.75, -0.66) |
| Central Europe | 10-14 years | 135.92 | 128.99 | -0.08 (-0.2, 0.04) | 14065.33 | 8108.93 | -0.76 (-0.8, -0.72) |
| Central Europe | 15-19 years | 130.86 | 125.53 | -0.07 (-0.2, 0.05) | 12580.68 | 7351.03 | -0.71 (-0.78, -0.63) |
| Central Europe | <20 years | 2918.92 | 810.85 | -0.72 (-0.76, -0.68) | 1125538.44 | 191013.69 | -0.72 (-0.78, -0.64) |
| Central Europe | Age-standardized | 3201.5 | 873.35 | - | - | - | - |
| Central Latin America | <5 years | 26743.1 | 8763 | -0.64 (-0.72, -0.55) | 6122670.41 | 1760522.84 | 0.02 (-0.13, 0.17) |
| Central Latin America | 5-9 years | 114.83 | 149.21 | 0.08 (-0.07, 0.23) | 24248.31 | 32095.91 | 0.05 (-0.1, 0.2) |
| Central Latin America | 10-14 years | 109.45 | 142.86 | 0.1 (-0.05, 0.24) | 21983.89 | 31262.33 | -0.69 (-0.71, -0.65) |
| Central Latin America | 15-19 years | 101.89 | 135.46 | 0.15 (0, 0.3) | 18538.58 | 29531.03 | -0.73 (-0.76, -0.71) |
| Central Latin America | <20 years | 7518.85 | 2173.2 | -0.68 (-0.75, -0.61) | 6187441.18 | 1853412.11 | -0.73 (-0.79, -0.66) |
| Central Latin America | Age-standardized | 6402.46 | 2373.80 | - | - | - | - |
| Central Sub-Saharan Africa | <5 years | 57208.47 | 27079.7 | -0.51 (-0.6, -0.42) | 6149888.67 | 5704745.06 | -0.47 (-0.53, -0.43) |
| Central Sub-Saharan Africa | 5-9 years | 39.54 | 114.78 | 0.54 (0.19, 1.04) | 3283.92 | 22863.53 | -0.54 (-0.59, -0.49) |
| Central Sub-Saharan Africa | 10-14 years | 35.82 | 103.89 | 0.54 (0.18, 0.98) | 2427.92 | 18383.61 | -0.68 (-0.74, -0.6) |
| Central Sub-Saharan Africa | 15-19 years | 32.2 | 89.57 | 0.52 (0.19, 0.9) | 1830.55 | 13327.07 | 0.06 (-0.15, 0.34) |
| Central Sub-Saharan Africa | <20 years | 19536.63 | 7829.34 | -0.58 (-0.65, -0.5) | 6157431.05 | 5759319.28 | 0.05 (-0.16, 0.3) |
| Central Sub-Saharan Africa | Age-standardized | 14260.56 | 7085.29 | - | - | - | - |
| East Asia | <5 years | 20780.52 | 2544.73 | -0.87 (-0.89, -0.84) | 24902005.46 | 2037656.94 | -0.46 (-0.54, -0.37) |
| East Asia | 5-9 years | 77.87 | 74.6 | -0.15 (-0.28, -0.03) | 84525.5 | 73425.37 | -0.75 (-0.79, -0.71) |
| East Asia | 10-14 years | 73.07 | 72.93 | -0.12 (-0.25, 0.01) | 77871.03 | 64803.27 | -0.08 (-0.2, 0.03) |
| East Asia | 15-19 years | 67.88 | 72.07 | -0.07 (-0.2,0.07) | 88605.57 | 55928.8 | -0.08 (-0.2, 0.04) |
| East Asia | <20 years | 5403.85 | 646.99 | -0.87 (-0.89, -0.84) | 25153007.56 | 2231814.37 | -0.07 (-0.2, 0.05) |
| East Asia | Age-standardized | 5033.32 | 712.91 | - | - | - | - |
| Eastern Europe | <5 years | 9946.62 | 2788.87 | -0.72 (-0.74, -0.69) | 1713646.79 | 282203.84 | -0.02 (-0.15, 0.13) |
| Eastern Europe | 5-9 years | 120.89 | 101.4 | 0.01 (-0.13, 0.17) | 21451.41 | 13066.18 | 0 (-0.14, 0.14) |
| Eastern Europe | 10-14 years | 116.04 | 98.12 | 0.02 (-0.13, 0.17) | 19119.65 | 12205.1 | -0.64 (-0.69, -0.59) |
| Eastern Europe | 15-19 years | 108.4 | 94.53 | 0.05 (-0.1, 0.2) | 17198.95 | 10128.96 | -0.69 (-0.73, -0.65) |
| Eastern Europe | <20 years | 2631.51 | 688.06 | -0.73 (-0.76, -0.71) | 1771416.8 | 317604.07 | -0.6 (-0.67, -0.53) |
| Eastern Europe | Age-standardized | 2620.52 | 794.52 | - | - | - | - |
| Eastern Sub-Saharan Africa | <5 years | 64637.24 | 32725.23 | -0.5 (-0.59, -0.38) | 23282274.1 | 20877514.3 | 0.05 (-0.19, 0.31) |
| Eastern Sub-Saharan Africa | 5-9 years | 58.87 | 173.75 | 0.73 (0.49, 1.01) | 17251.94 | 103320.96 | -0.6 (-0.67, -0.51) |
| Eastern Sub-Saharan Africa | 10-14 years | 54.37 | 164.76 | 0.79 (0.54, 1.05) | 13615 | 90903 | -0.71 (-0.77, -0.65) |
| Eastern Sub-Saharan Africa | 15-19 years | 50.22 | 147.34 | 0.81 (0.58, 1.1) | 10176.24 | 72424.73 | -0.73 (-0.77, -0.68) |
| Eastern Sub-Saharan Africa | <20 years | 21081.88 | 9290.68 | -0.57 (-0.65, -0.47) | 23323317.27 | 21144162.99 | -0.09 (-0.21, 0.04) |
| Eastern Sub-Saharan Africa | Age-standardized | 17111.11 | 8590.29 | - | - | - | - |
| High-income Asia Pacific | <5 years | 3715.29 | 916.2 | -0.76 (-0.79, -0.73) | 380845.48 | 59114.6 | -0.43 (-0.54, -0.32) |
| High-income Asia Pacific | 5-9 years | 93.99 | 92.7 | -0.03 (-0.18, 0.12) | 11177.87 | 7228.53 | -0.58 (-0.66, -0.5) |
| High-income Asia Pacific | 10-14 years | 91.97 | 91.51 | -0.02 (-0.15, 0.13) | 12047.75 | 7481.59 | -0.5 (-0.6, -0.39) |
| High-income Asia Pacific | 15-19 years | 89.04 | 89.92 | 0 (-0.14, 0.14) | 13481.75 | 7520.76 | -0.43 (-0.54, -0.29) |
| High-income Asia Pacific | <20 years | 828.74 | 264.2 | -0.69 (-0.73, -0.65) | 417552.85 | 81345.49 | -0.57 (-0.65, -0.47) |
| High-income Asia Pacific | Age-standardized | 1066.33 | 304.88 | - | - | - | - |
| High-income North America | <5 years | 8096.99 | 4113.4 | -0.45 (-0.5, -0.39) | 1740632.91 | 843187.76 | -0.24 (-0.38, -0.04) |
| High-income North America | 5-9 years | 198.87 | 191.03 | -0.06 (-0.14, 0.04) | 40782.02 | 41775.16 | -0.23 (-0.38, -0.06) |
| High-income North America | 10-14 years | 193.83 | 186.88 | -0.06 (-0.15, 0.04) | 37619.33 | 43454.18 | -0.22 (-0.37, -0.01) |
| High-income North America | 15-19 years | 184.18 | 180.17 | -0.05 (-0.14, 0.06) | 36910.17 | 43130.79 | -0.68 (-0.75, -0.61) |
| High-income North America | <20 years | 2278.57 | 1084.83 | -0.49 (-0.54, -0.44) | 1855944.44 | 971547.89 | -0.74 (-0.8, -0.68) |
| High-income North America | Age-standardized | 2075.97 | 1202.61 | - | - | - | - |
| North Africa and Middle East | <5 years | 49314.3 | 11847.47 | -0.73 (-0.77, -0.68) | 26305128.57 | 7243200.96 | -0.11 (-0.24, 0.04) |
| North Africa and Middle East | 5-9 years | 161.96 | 205.84 | -0.09 (-0.21, 0.04) | 77754.4 | 130096.07 | -0.45 (-0.53, -0.36) |
| North Africa and Middle East | 10-14 years | 152.82 | 195.74 | -0.09 (-0.22, 0.04) | 64751.63 | 115454.72 | -0.49 (-0.57, -0.4) |
| North Africa and Middle East | 15-19 years | 141.46 | 182.93 | -0.08 (-0.2, 0.05) | 51736.47 | 97265.35 | -0.72 (-0.74, -0.69) |
| North Africa and Middle East | <20 years | 14697.8 | 3207.73 | -0.75 (-0.79, -0.71) | 26499371.07 | 7586017.1 | 0.01 (-0.13, 0.17) |
| North Africa and Middle East | Age-standardized | 11406.79 | 3210.93 | - | - | - | - |
| Oceania | <5 years | 28474.25 | 22300.68 | -0.19 (-0.34, 0) | 279949.09 | 431395.78 | -0.7 (-0.77, -0.62) |
| Oceania | 5-9 years | 114.48 | 197.6 | -0.12 (-0.27, 0.06) | 988.25 | 3294.86 | -0.52 (-0.61, -0.43) |
| Oceania | 10-14 years | 106.69 | 184.05 | -0.13 (-0.29, 0.03) | 833.53 | 2722.04 | -0.48 (-0.57, -0.37) |
| Oceania | 15-19 years | 96.8 | 170 | -0.12 (-0.27, 0.07) | 660.68 | 2219.42 | -0.62 (-0.68, -0.54) |
| Oceania | <20 years | 8532.13 | 6883.90 | -0.17 (-0.33, 0.01) | 282431.56 | 439632.11 | -0.51 (-0.6, -0.42) |
| Oceania | Age-standardized | 7242.45 | 5908.53 | - | - | - | - |
| South Asia | <5 years | 77191.34 | 35127.59 | -0.52 (-0.6, -0.43) | 124873428.63 | 55710160.56 | -0.31 (-0.45, -0.14) |
| South Asia | 5-9 years | 156.77 | 527.07 | 0.27 (0.16, 0.4) | 235309.7 | 897740.83 | -0.21 (-0.3, -0.08) |
| South Asia | 10-14 years | 140.16 | 510.59 | 0.35 (0.23, 0.49) | 178159.52 | 909361.63 | -0.2 (-0.3, -0.08) |
| South Asia | 15-19 years | 124.48 | 479.87 | 0.44 (0.31, 0.58) | 135780.45 | 846845.8 | -0.16 (-0.26, -0.04) |
| South Asia | <20 years | 22884.84 | 8539.07 | -0.6 (-0.67, -0.53) | 125422678.31 | 58364108.81 | -0.26 (-0.4, -0.1) |
| South Asia | Age-standardized | 19183.39 | 9467.09 | - | - | - | - |
| Southeast Asia | <5 years | 35270.8 | 13090.61 | -0.58 (-0.66, -0.51) | 20980435.22 | 7367990.94 | -0.7 (-0.74, -0.66) |
| Southeast Asia | 5-9 years | 101.23 | 161.25 | 0.03 (-0.08, 0.15) | 58865.48 | 93986.02 | -0.72 (-0.76, -0.68) |
| Southeast Asia | 10-14 years | 95.56 | 154.8 | 0.05 (-0.06, 0.17) | 52173.63 | 89913.12 | -0.19 (-0.34, 0) |
| Southeast Asia | 15-19 years | 89.24 | 145.43 | 0.07 (-0.05, 0.19) | 43775.85 | 82337.5 | -0.12 (-0.27, 0.06) |
| Southeast Asia | <20 years | 9551.09 | 3329.78 | -0.61 (-0.67, -0.54) | 21135250.18 | 7634227.59 | -0.13 (-0.29, 0.03) |
| Southeast Asia | Age-standardized | 8266.24 | 9467.09 | - | - | - | - |
| Southern Latin America | <5 years | 17447.43 | 5410.55 | -0.68 (-0.74, -0.6) | 900155.6 | 231488.34 | -0.54 (-0.61, -0.47) |
| Southern Latin America | 5-9 years | 122.66 | 163.13 | 0.06 (-0.15, 0.34) | 6122.96 | 8414.78 | -0.61 (-0.67, -0.54) |
| Southern Latin America | 10-14 years | 119.98 | 159.6 | 0.05 (-0.16, 0.3) | 5733.81 | 8073.65 | -0.47 (-0.55, -0.38) |
| Southern Latin America | 15-19 years | 114.91 | 153.21 | 0.05 (-0.19, 0.31) | 5105.75 | 7681.94 | -0.11 (-0.24, 0.04) |
| Southern Latin America | <20 years | 4733.95 | 1310.42 | -0.71 (-0.77, -0.65) | 917118.12 | 255658.71 | -0.11 (-0.25, 0.02) |
| Southern Latin America | Age-standardized | 4462.55 | 1518.10 | - | - | - | - |
| Southern Sub-Saharan Africa | <5 years | 39033.61 | 27462.82 | -0.25 (-0.38, -0.09) | 2800239.18 | 2205021.07 | 0.08 (-0.07, 0.23) |
| Southern Sub-Saharan Africa | 5-9 years | 170.85 | 200.43 | 0.4 (0.21, 0.64) | 11634.29 | 16346.09 | 0.1 (-0.05, 0.24) |
| Southern Sub-Saharan Africa | 10-14 years | 157.02 | 185.03 | 0.39 (0.2, 0.62) | 10036.19 | 14582.7 | 0.15 (0, 0.3) |
| Southern Sub-Saharan Africa | 15-19 years | 143.79 | 165.15 | 0.4 (0.2, 0.62) | 8386.69 | 11888.34 | -0.57 (-0.66, -0.48) |
| Southern Sub-Saharan Africa | <20 years | 10799.39 | 7189.75 | -0.31 (-0.43, -0.17) | 2830296.35 | 2247838.2 | -0.68 (-0.75, -0.61) |
| Southern Sub-Saharan Africa | Age-standardized | 9587.40 | 7244.37 | - | - | - | - |
| Tropical Latin America | <5 years | 37530.98 | 8621.21 | -0.71 (-0.77, -0.63) | 6588651.65 | 1483503.91 | -0.36 (-0.48, -0.21) |
| Tropical Latin America | 5-9 years | 100.76 | 204.57 | 0.81 (0.53, 1.07) | 18750.37 | 34180.54 | -0.57 (-0.62, -0.53) |
| Tropical Latin America | 10-14 years | 96.55 | 198.05 | 0.83 (0.55, 1.07) | 17167.37 | 32237.41 | -0.02 (-0.13, 0.12) |
| Tropical Latin America | 15-19 years | 90.69 | 187.05 | 0.85 (0.6, 1.11) | 14228.19 | 30666.5 | -0.01 (-0.13, 0.13) |
| Tropical Latin America | <20 years | 9533.92 | 2373.7 | -0.68 (-0.74, -0.6) | 6638797.59 | 1580588.36 | 0.01 (-0.11, 0.12) |
| Tropical Latin America | Age-standardized | 7660.62 | 2377.21 | - | - | - | - |
| Western Europe | <5 years | 5545.59 | 2293.74 | -0.57 (-0.62, -0.53) | 1273209.36 | 486943.27 | -0.87 (-0.89, -0.84) |
| Western Europe | 5-9 years | 125.8 | 121.22 | -0.02 (-0.13, 0.12) | 29587.52 | 27841.22 | -0.58 (-0.66, -0.51) |
| Western Europe | 10-14 years | 123.59 | 117.61 | -0.01 (-0.13, 0.13) | 30368.44 | 28132.53 | 0.03 (-0.08, 0.15) |
| Western Europe | 15-19 years | 119.55 | 115.74 | 0.01 (-0.11, 0.12) | 32701.17 | 27306.74 | 0.05 (-0.06, 0.17) |
| Western Europe | <20 years | 1388.01 | 621.76 | -0.54 (-0.59, -0.49) | 1365866.49 | 570223.75 | 0.07 (-0.05, 0.19) |
| Western Europe | Age-standardized | 1478.18 | 681.38 | - | - | - | - |
| Western Sub-Saharan Africa | <5 years | 76762.2 | 48017.26 | -0.4 (-0.49, -0.29) | 27383394.97 | 38393688.15 | -0.09 (-0.22, 0.04) |
| Western Sub-Saharan Africa | 5-9 years | 43.22 | 121.47 | 0.52 (0.35, 0.72) | 12347.61 | 86912.16 | -0.08 (-0.2, 0.05) |
| Western Sub-Saharan Africa | 10-14 years | 39.74 | 115.09 | 0.58 (0.41, 0.8) | 9366.98 | 72798.97 | -0.68 (-0.73, -0.63) |
| Western Sub-Saharan Africa | 15-19 years | 36.85 | 105.08 | 0.63 (0.46, 0.84) | 7193.93 | 56546.77 | -0.75 (-0.79, -0.71) |
| Western Sub-Saharan Africa | <20 years | 25537.57 | 14375.79 | -0.46 (-0.54, -0.36) | 27412303.49 | 38609946.04 | -0.64 (-0.72, -0.55) |
| Western Sub-Saharan Africa | Age-standardized | 20684.56 | 12512.53 |  | - | - | - |
| **Deaths** |  |  |  |  |  |  |  |
| Andean Latin America | <5 years | 386.57 | 91.76 | -0.73 (-0.79, -0.66) | 21054.96 | 5648.6 | -0.75 (-0.79, -0.71) |
| Andean Latin America | <20 years | 110.03 | 23.86 | -0.75 (-0.81, -0.69) | 21054.96 | 5648.6 | -0.31 (-0.45, -0.14) |
| Andean Latin America | Age-standardized | 88.15 | 23.75 | - | - | - | - |
| Australasia | <5 years | 63.81 | 24.15 | -0.62 (-0.68, -0.54) | 984.51 | 438.6 | -0.77 (-0.8, -0.74) |
| Australasia | <20 years | 15.69 | 5.82 | -0.63 (-0.69, -0.55) | 984.51 | 438.6 | -0.19 (-0.34, 0) |
| Australasia | Age-standardized | 16.37 | 6.25 | - | - | - | - |
| Caribbean | <5 years | 365.78 | 259.98 | -0.31 (-0.45, -0.14) | 15139.92 | 10056.35 | -0.59 (-0.66, -0.51) |
| Caribbean | <20 years | 100.34 | 65.89 | -0.36 (-0.49, -0.21) | 15139.92 | 10056.35 | -0.56 (-0.64, -0.48) |
| Caribbean | Age-standardized | 97.53 | 67.29 | - | - | - | - |
| Central Asia | <5 years | 272.36 | 137.69 | -0.47 (-0.55, -0.38) | 25817.7 | 13764.66 | -0.82 (-0.85, -0.78) |
| Central Asia | <20 years | 81.85 | 39.75 | -0.49 (-0.57, -0.4) | 25817.7 | 13764.66 | -0.88 (-0.9, -0.86) |
| Central Asia | Age-standardized | 67.50 | 35.64 | - | - | - | - |
| Central Europe | <5 years | 135.17 | 32.04 | -0.76 (-0.8, -0.71) | 12075.38 | 1789.41 | -0.18 (-0.34, 0.01) |
| Central Europe | <20 years | 31.32 | 7.6 | -0.75 (-0.79, -0.71) | 12075.38 | 1789.41 | -0.17 (-0.33, 0.02) |
| Central Europe | Age-standardized | 34.05 | 8.29 | - | - | - | - |
| Central Latin America | <5 years | 299.57 | 95.7 | -0.64 (-0.72, -0.55) | 68583.89 | 19226.49 | -0.6 (-0.67, -0.52) |
| Central Latin America | <20 years | 83.34 | 22.54 | -0.7 (-0.76, -0.62) | 68583.89 | 19226.49 | -0.63 (-0.69, -0.55) |
| Central Latin America | Age-standardized | 69.68 | 24.77 | - | - | - | - |
| Central Sub-Saharan Africa | <5 years | 642.65 | 299.27 | -0.51 (-0.6, -0.42) | 69084.81 | 63046.18 | -0.6 (-0.69, -0.5) |
| Central Sub-Saharan Africa | <20 years | 219.2 | 85.71 | -0.58 (-0.66,-0.5) | 69084.81 | 63046.18 | -0.7 (-0.76, -0.62) |
| Central Sub-Saharan Africa | Age-standardized | 157.55 | 77.46 | - | - | - | - |
| East Asia | <5 years | 232.95 | 27.46 | -0.87 (-0.9, -0.84) | 279157.7 | 21985.39 | -0.59 (-0.64, -0.54) |
| East Asia | <20 years | 59.97 | 6.37 | -0.88 (-0.9, -0.86) | 279157.7 | 21985.39 | -0.87 (-0.9, -0.84) |
| East Asia | Age-standardized | 54.98 | 7.11 | - | - | - | - |
| Eastern Europe | <5 years | 110.6 | 29.95 | -0.72 (-0.75, -0.69) | 19054.54 | 3030.34 | -0.47 (-0.55, -0.38) |
| Eastern Europe | <20 years | 28.31 | 6.56 | -0.76 (-0.79, -0.74) | 19054.54 | 3030.34 | -0.47 (-0.55, -0.37) |
| Eastern Europe | Age-standardized | 28.07 | 7.75 | - | - | - | - |
| Eastern Sub-Saharan Africa | <5 years | 726.15 | 361.49 | -0.5 (-0.6, -0.39) | 261557.7 | 230619.42 | -0.68 (-0.75, -0.6) |
| Eastern Sub-Saharan Africa | <20 years | 236.42 | 101.33 | -0.57 (-0.65, -0.47) | 261557.7 | 230619.42 | -0.64 (-0.72, -0.55) |
| Eastern Sub-Saharan Africa | Age-standardized | 188.99 | 93.56 | - | - | - | - |
| High-income Asia Pacific | <5 years | 40.78 | 9.16 | -0.78 (-0.81, -0.75) | 4180.38 | 590.73 | -0.49 (-0.57, -0.4) |
| High-income Asia Pacific | <20 years | 8.3 | 1.92 | -0.77 (-0.8, -0.74) | 4180.38 | 590.73 | -0.72 (-0.75, -0.69) |
| High-income Asia Pacific | Age-standardized | 10.82 | 2.37 | - | - | - | - |
| High-income North America | <5 years | 88.92 | 43.64 | -0.46 (-0.52, -0.4) | 19114.69 | 8945.49 | -0.62 (-0.69, -0.55) |
| High-income North America | <20 years | 23.47 | 9.99 | -0.53 (-0.58, -0.48) | 19114.69 | 8945.49 | -0.62 (-0.68, -0.54) |
| High-income North America | Age-standardized | 20.88 | 11.29 | - | - | - | - |
| North Africa and Middle East | <5 years | 553.08 | 129.29 | -0.73 (-0.77, -0.68) | 295020.12 | 79044.47 | -0.27 (-0.42, -0.09) |
| North Africa and Middle East | <20 years | 163.63 | 33.42 | -0.76 (-0.8, -0.72) | 295020.12 | 79044.47 | -0.36 (-0.49, -0.21) |
| North Africa and Middle East | Age-standardized | 124.35 | 33.46 | - | - | - | - |
| Oceania | <5 years | 318.97 | 245.45 | -0.19 (-0.34, 0) | 3135.96 | 4748.09 | -0.73 (-0.76, -0.7) |
| Oceania | <20 years | 94.74 | 74.35 | -0.17 (-0.33, 0.02) | 3135.96 | 4748.09 | -0.76 (-0.79, -0.74) |
| Oceania | Age-standardized | 78.08 | 63.53 | - | - | - | - |
| South Asia | <5 years | 866.16 | 383.94 | -0.52 (-0.61, -0.43) | 1401190.68 | 608899.82 | -0.53 (-0.58, -0.48) |
| South Asia | <20 years | 255.66 | 89.09 | -0.62 (-0.68, -0.54) | 1401190.68 | 608899.82 | -0.64 (-0.72, -0.55) |
| South Asia | Age-standardized | 208.49 | 99.37 | - | - | - | - |
| Southeast Asia | <5 years | 395.64 | 143.67 | -0.59 (-0.66, -0.51) | 235343.31 | 80862.27 | -0.78 (-0.81, -0.75) |
| Southeast Asia | <20 years | 106.35 | 35.27 | -0.62 (-0.69, -0.55) | 235343.31 | 80862.27 | -0.77 (-0.8, -0.74) |
| Southeast Asia | Age-standardized | 90.18 | 37.18 | - | - | - | - |
| Southern Latin America | <5 years | 194.99 | 58.37 | -0.68 (-0.75, -0.6) | 10060 | 2497.24 | -0.76 (-0.8, -0.71) |
| Southern Latin America | <20 years | 51.93 | 12.8 | -0.74 (-0.79, -0.67) | 10060 | 2497.24 | -0.76 (-0.8, -0.71) |
| Southern Latin America | Age-standardized | 47.93 | 15.11 | - | - | - | - |
| Southern Sub-Saharan Africa | <5 years | 436.6 | 302.82 | -0.25 (-0.39, -0.09) | 31321.49 | 24313.86 | -0.74 (-0.79, -0.67) |
| Southern Sub-Saharan Africa | <20 years | 119.51 | 77.77 | -0.32 (-0.44, -0.17) | 31321.49 | 24313.86 | -0.73 (-0.79, -0.66) |
| Southern Sub-Saharan Africa | Age-standardized | 104.95 | 78.38 | - | - | - | - |
| Tropical Latin America | <5 years | 421.22 | 93.53 | -0.71 (-0.78, -0.63) | 73945.53 | 16094.4 | -0.46 (-0.52, -0.4) |
| Tropical Latin America | <20 years | 106.19 | 24.17 | -0.7 (-0.77, -0.62) | 73945.53 | 16094.4 | -0.42 (-0.49, -0.36) |
| Tropical Latin America | Age-standardized | 83.92 | 24.21 | - | - | - | - |
| Western Europe | <5 years | 61.01 | 24.14 | -0.59 (-0.64, -0.54) | 14008.04 | 5124.72 | -0.59 (-0.64, -0.54) |
| Western Europe | <20 years | 14.24 | 5.59 | -0.59 (-0.64, -0.54) | 14008.04 | 5124.72 | -0.57 (-0.62, -0.52) |
| Western Europe | Age-standardized | 15.10 | 6.25 | - | - | - | - |
| Western Sub-Saharan Africa | <5 years | 862.76 | 532.04 | -0.4 (-0.49, -0.29) | 307774.02 | 425406.01 | -0.7 (-0.77, -0.63) |
| Western Sub-Saharan Africa | <20 years | 286.73 | 158.39 | -0.46 (-0.54, -0.37) | 307774.02 | 425406.01 | -0.75 (-0.81, -0.69) |
| Western Sub-Saharan Africa | Age-standardized | 229.06 | 137.70 | - | - | - | - |
| **YLDs (Years Lived with Disability)** | |  |  |  |  |  |  |
| Andean Latin America | <5 years | 137.11 | 144.94 | -0.29 (-0.41, -0.14) | 7467.57 | 8922.44 | 0.02 (-0.11, 0.16) |
| Andean Latin America | 5-9 years | 114.04 | 136.17 | -0.24 (-0.38, -0.04) | 5671.66 | 8295.72 | -0.16 (-0.33, 0.03) |
| Andean Latin America | 10-14 years | 107.86 | 131.45 | -0.23 (-0.38, -0.06) | 4988.12 | 7685.36 | -0.18 (-0.36, 0.06) |
| Andean Latin America | 15-19 years | 99.96 | 123.43 | -0.22 (-0.37,-0.01) | 4089.75 | 6885.88 | -0.17 (-0.36, 0.06) |
| Andean Latin America | <20 years | 116.1 | 134.28 | -0.25 (-0.37, -0.12) | 22217.1 | 31789.41 | -0.17 (-0.37, 0.09) |
| Andean Latin America | Age-standardized | 178.56 | 134.23 | - | - | - | - |
| Australasia | <5 years | 160.93 | 144.55 | -0.16 (-0.33, 0.03) | 2482.83 | 2625.01 | 0.54 (0.18, 0.98) |
| Australasia | 5-9 years | 164.92 | 147.24 | -0.18 (-0.36, 0.06) | 2519.11 | 2878.61 | 0.52 (0.19, 0.9) |
| Australasia | 10-14 years | 161.15 | 144.37 | -0.17 (-0.36, 0.06) | 2444.23 | 2829.72 | -0.42 (-0.52, -0.31) |
| Australasia | 15-19 years | 154.78 | 140.12 | -0.17 (-0.37, 0.09) | 2612.42 | 2537.15 | -0.58 (-0.65, -0.5) |
| Australasia | <20 years | 160.3 | 144.14 | -0.17 (-0.32, 0) | 10058.6 | 10870.5 | -0.5 (-0.59, -0.38) |
| Australasia | Age-standardized | 173.56 | 143.71 | - | - | - | - |
| Caribbean | <5 years | 196.89 | 249.42 | -0.19 (-0.28, -0.08) | 8149.66 | 9648 | 0.15 (0, 0.3) |
| Caribbean | 5-9 years | 179.17 | 234.29 | -0.21 (-0.3, -0.08) | 6675.84 | 9032.43 | 0.12 (-0.02, 0.24) |
| Caribbean | 10-14 years | 172.67 | 224.91 | -0.2 (-0.3, -0.08) | 6127.2 | 8505.11 | 0.07 (-0.06, 0.2) |
| Caribbean | 15-19 years | 160.43 | 211.17 | -0.16 (-0.26, -0.04) | 5895.42 | 7934.51 | 0.21 (0.06, 0.36) |
| Caribbean | <20 years | 177.94 | 230.11 | -0.19 (-0.28, -0.1) | 26848.12 | 35120.05 | 0.73 (0.49, 1.01) |
| Caribbean | Age-standardized | 285.21 | 230.34 | - | - | - | - |
| Central Asia | <5 years | 121.53 | 113.46 | -0.16 (-0.27, -0.03) | 11520.37 | 11342.51 | -0.44 (-0.53, -0.34) |
| Central Asia | 5-9 years | 104.46 | 111.11 | -0.11 (-0.24, 0.04) | 8605.28 | 10476.61 | -0.6 (-0.67, -0.53) |
| Central Asia | 10-14 years | 99.65 | 106.98 | -0.11 (-0.25, 0.02) | 7213.41 | 8825.45 | -0.71 (-0.77, -0.63) |
| Central Asia | 15-19 years | 93.45 | 100.55 | -0.11 (-0.24, 0.04) | 6154.5 | 6986.78 | 0.81 (0.53, 1.07) |
| Central Asia | <20 years | 106.19 | 108.68 | -0.13 (-0.23, -0.01) | 33493.56 | 37631.35 | 0.83 (0.55, 1.07) |
| Central Asia | Age-standardized | 123.66 | 108.20 | - | - | - | - |
| Central Europe | <5 years | 144.04 | 124.13 | -0.09 (-0.2, 0.02) | 12868.04 | 6933.43 | -0.45 (-0.5, -0.39) |
| Central Europe | 5-9 years | 138.9 | 131.33 | -0.08 (-0.2, 0.03) | 13424.61 | 7655.19 | -0.06 (-0.14, 0.04) |
| Central Europe | 10-14 years | 135.92 | 128.99 | -0.08 (-0.2, 0.04) | 14065.33 | 8108.93 | -0.06 (-0.15, 0.04) |
| Central Europe | 15-19 years | 130.86 | 125.53 | -0.07 (-0.2, 0.05) | 12580.68 | 7351.03 | -0.05 (-0.14, 0.06) |
| Central Europe | <20 years | 137.29 | 127.56 | -0.08 (-0.19, 0.02) | 52938.66 | 30048.57 | -0.3 5(-0.41, -0.3) |
| Central Europe | Age-standardized | 138.45 | 127.49 | - | - | - | - |
| Central Latin America | <5 years | 132.03 | 154.46 | 0.02 (-0.1, 0.14) | 30226.44 | 31031.64 | -0.07 (-0.2, 0.05) |
| Central Latin America | 5-9 years | 114.83 | 149.21 | 0.08 (-0.07, 0.23) | 24248.31 | 32095.91 | -0.04 (-0.15, 0.06) |
| Central Latin America | 10-14 years | 109.45 | 142.86 | 0.1 (-0.05, 0.24) | 21983.89 | 31262.33 | -0.08 (-0.19, 0.02) |
| Central Latin America | 15-19 years | 101.89 | 135.46 | 0.15 (0, 0.3) | 18538.58 | 29531.03 | -0.1 (-0.24, 0.05) |
| Central Latin America | <20 years | 115.44 | 145.3 | 0.07 (-0.06, 0.2) | 94997.21 | 123920.91 | -0.12 (-0.27, 0.06) |
| Central Latin America | Age-standardized | 134.79 | 145.70 | - | - | - | - |
| Central Sub-Saharan Africa | <5 years | 110.6 | 161.10 | 0.06 (-0.12, 0.28) | 11889.28 | 33938.66 | -0.01 (-0.13, 0.13) |
| Central Sub-Saharan Africa | 5-9 years | 39.54 | 114.78 | 0.54 (0.19, 1.04) | 3283.92 | 22863.53 | 0.01 (-0.11, 0.12) |
| Central Sub-Saharan Africa | 10-14 years | 35.82 | 103.89 | 0.54 (0.18, 0.98) | 2427.92 | 18383.61 | 0.01 (-0.09, 0.12) |
| Central Sub-Saharan Africa | 15-19 years | 32.2 | 89.57 | 0.52 (0.19, 0.9) | 1830.55 | 13327.07 | -0.01 (-0.1, 0.1) |
| Central Sub-Saharan Africa | <20 years | 61.65 | 120.33 | 0.25 (0.03, 0.51) | 19431.66 | 88512.87 | -0.29 (-0.39, -0.18) |
| Central Sub-Saharan Africa | Age-standardized | 88.95 | 118.12 | - | - | - | - |
| East Asia | <5 years | 93.85 | 75.7 | -0.29 (-0.39, -0.18) | 112466.36 | 60614.26 | -0.46 (-0.54, -0.36) |
| East Asia | 5-9 years | 77.87 | 74.6 | -0.15 (-0.28, -0.03) | 84525.5 | 73425.37 | -0.25 (-0.38, -0.09) |
| East Asia | 10-14 years | 73.07 | 72.93 | -0.12 (-0.25, 0.01) | 77871.03 | 64803.27 | 0.4 (0.21, 0.64) |
| East Asia | 15-19 years | 67.88 | 72.07 | -0.07 (-0.2, 0.07) | 88605.57 | 55928.8 | 0.39 (0.2, 0.62) |
| East Asia | <20 years | 78.09 | 73.86 | -0.17 (-0.28, -0.06) | 363468.46 | 254771.69 | 0.4 (0.2, 0.62) |
| East Asia | Age-standardized | 89.30 | 73.87 | - | - | - | - |
| Eastern Europe | <5 years | 120.7 | 94.84 | -0.01 (-0.14, 0.13) | 20794.04 | 9596.54 | 0.85 (0.6, 1.11) |
| Eastern Europe | 5-9 years | 120.89 | 101.4 | 0.01 (-0.13, 0.17) | 21451.41 | 13066.18 | -0.68 (-0.74, -0.61) |
| Eastern Europe | 10-14 years | 116.04 | 98.12 | 0.02 (-0.13, 0.17) | 19119.65 | 12205.1 | -0.68 (-0.74, -0.6) |
| Eastern Europe | 15-19 years | 108.4 | 94.53 | 0.05 (-0.1, 0.2) | 17198.95 | 10128.96 | -0.51 (-0.6, -0.42) |
| Eastern Europe | <20 years | 116.71 | 97.48 | 0.02 (-0.11, 0.16) | 78564.05 | 44996.77 | 0.54 (0.19, 1.04) |
| Eastern Europe | Age-standardized | 95.48 | 97.24 | - | - | - | - |
| Eastern Sub-Saharan Africa | <5 years | 124.78 | 209.03 | 0.21 (0.06, 0.36) | 44944.27 | 133353.69 | -0.13 (-0.29, 0.03) |
| Eastern Sub-Saharan Africa | 5-9 years | 58.87 | 173.75 | 0.73 (0.49, 1.01) | 17251.94 | 103320.96 | -0.12 (-0.27, 0.07) |
| Eastern Sub-Saharan Africa | 10-14 years | 54.37 | 164.76 | 0.79 (0.54, 1.05) | 13615 | 90903 | -0.1 (-0.21, 0.03) |
| Eastern Sub-Saharan Africa | 15-19 years | 50.22 | 147.34 | 0.81 (0.58, 1.1) | 10176.24 | 72424.73 | -0.11 (-0.24, 0.02) |
| Eastern Sub-Saharan Africa | <20 years | 77.72 | 175.76 | 0.48 (0.31, 0.67) | 85987.45 | 400002.38 | -0.16 (-0.27, -0.03) |
| Eastern Sub-Saharan Africa | Age-standardized | 112.64 | 174.34 | - | - | - | - |
| High-income Asia Pacific | <5 years | 94.97 | 93.05 | -0.03 (-0.15, 0.08) | 9735.46 | 6004.04 | -0.19 (-0.33, -0.02) |
| High-income Asia Pacific | 5-9 years | 93.99 | 92.7 | -0.03 (-0.18, 0.12) | 11177.87 | 7228.53 | -0.31 (-0.43, -0.17) |
| High-income Asia Pacific | 10-14 years | 91.97 | 91.51 | -0.02 (-0.15, 0.13) | 12047.75 | 7481.59 | -0.09 (-0.2, 0.02) |
| High-income Asia Pacific | 15-19 years | 89.04 | 89.92 | 0 (-0.14, 0.14) | 13481.75 | 7520.76 | -0.08 (-0.2, 0.03) |
| High-income Asia Pacific | <20 years | 92.18 | 91.7 | -0.02 (-0.13, 0.09) | 46442.83 | 28234.92 | -0.08 (-0.2, 0.04) |
| High-income Asia Pacific | Age-standardized | 93.83 | 91.83 | - | - | - | - |
| High-income North America | <5 years | 201.02 | 188.34 | -0.06 (-0.14, 0.02) | 43214.8 | 38607.22 | -0.02 (-0.13, 0.09) |
| High-income North America | 5-9 years | 198.87 | 191.03 | -0.06 (-0.14, 0.04) | 40782.02 | 41775.16 | 0.02 (-0.1, 0.14) |
| High-income North America | 10-14 years | 193.83 | 186.88 | -0.06 (-0.15, 0.04) | 37619.33 | 43454.18 | 0.08 (-0.07, 0.23) |
| High-income North America | 15-19 years | 184.18 | 180.17 | -0.05 (-0.14, 0.06) | 36910.17 | 43130.79 | 0.1 (-0.05, 0.24) |
| High-income North America | <20 years | 194.63 | 186.43 | -0.06 (-0.13, 0.03) | 158526.32 | 166967.35 | 0.79 (0.54, 1.05) |
| High-income North America | Age-standardized | 93.83 | 91.83 | - | - | - | - |
| North Africa and Middle East | <5 years | 197.95 | 219.32 | -0.15 (-0.25, -0.05) | 105589.15 | 134084.52 | 0.07 (-0.05, 0.19) |
| North Africa and Middle East | 5-9 years | 161.96 | 205.84 | -0.09 (-0.21, 0.04) | 77754.4 | 130096.07 | 0.05 (-0.05, 0.14) |
| North Africa and Middle East | 10-14 years | 152.82 | 195.74 | -0.09 (-0.22, 0.04) | 64751.63 | 115454.72 | 0.02 (-0.08, 0.11) |
| North Africa and Middle East | 15-19 years | 141.46 | 182.93 | -0.08 (-0.2, 0.05) | 51736.47 | 97265.35 | -0.01 (-0.12, 0.09) |
| North Africa and Middle East | <20 years | 166.3 | 201.66 | -0.11 (-0.22, 0) | 299831.65 | 476900.66 | -0.02 (-0.13, 0.12) |
| North Africa and Middle East | Age-standardized | 224.59 | 201.29 | - | - | - | - |
| Oceania | <5 years | 142.25 | 221.75 | -0.1 (-0.24, 0.05) | 1398.57 | 4289.64 | -0.49 (-0.54, -0.44) |
| Oceania | 5-9 years | 114.48 | 197.6 | -0.12 (-0.27, 0.06) | 988.25 | 3294.86 | -0.52 (-0.6, -0.43) |
| Oceania | 10-14 years | 106.69 | 184.05 | -0.13 (-0.29, 0.03) | 833.53 | 2722.04 | 0.27 (0.16, 0.4) |
| Oceania | 15-19 years | 96.8 | 170 | -0.12 (-0.27, 0.07) | 660.68 | 2219.42 | 0.35 (0.23, 0.49) |
| Oceania | <20 years | 117.24 | 196.14 | -0.11 (-0.24, 0.02) | 3881.04 | 12525.97 | 0.44 (0.31, 0.58) |
| Oceania | Age-standardized | 219.10 | 193.98 | - | - | - | - |
| South Asia | <5 years | 233.77 | 584.7 | 0.03 (-0.05, 0.11) | 378178.02 | 927290.05 | -0.15 (-0.28, -0.03) |
| South Asia | 5-9 years | 156.77 | 527.07 | 0.27 (0.16, 0.4) | 235309.7 | 897740.83 | -0.12 (-0.25, 0.01) |
| South Asia | 10-14 years | 140.16 | 510.59 | 0.35 (0.23, 0.49) | 178159.52 | 909361.63 | -0.07 (-0.2, 0.07) |
| South Asia | 15-19 years | 124.48 | 479.87 | 0.44 (0.31, 0.58) | 135780.45 | 846845.8 | -0.07 (-0.2, 0.04) |
| South Asia | <20 years | 169.22 | 523.96 | 0.21 (0.11, 0.32) | 927427.69 | 3581238.3 | -0.17 (-0.28, -0.06) |
| South Asia | Age-standardized | 425.40 | 526.58 | - | - | - | - |
| Southeast Asia | <5 years | 122.46 | 165.54 | -0.04 (-0.14, 0.05) | 72845.3 | 93171.43 | 0.73 (0.49, 1.01) |
| Southeast Asia | 5-9 years | 101.23 | 161.25 | 0.03 (-0.08, 0.15) | 58865.48 | 93986.02 | 0.79 (0.54, 1.05) |
| Southeast Asia | 10-14 years | 95.56 | 154.8 | 0.05 (-0.06, 0.17) | 52173.63 | 89913.12 | 0.81 (0.58, 1.1) |
| Southeast Asia | 15-19 years | 89.24 | 145.43 | 0.07 (-0.05, 0.19) | 43775.85 | 82337.5 | -0.42 (-0.52, -0.28) |
| Southeast Asia | <20 years | 102.88 | 156.76 | 0.02 (-0.08, 0.11) | 227660.26 | 359408.07 | -0.57 (-0.65, -0.47) |
| Southeast Asia | Age-standardized | 153.47 | 156.94 | - | - | - | - |
| Southern Latin America | <5 years | 126.78 | 160.17 | 0.05 (-0.13, 0.25) | 6540.94 | 6852.78 | -0.11 (-0.24, 0.04) |
| Southern Latin America | 5-9 years | 122.66 | 163.13 | 0.06 (-0.15, 0.34) | 6122.96 | 8414.78 | -0.11 (-0.25, 0.02) |
| Southern Latin America | 10-14 years | 119.98 | 159.6 | 0.05 (-0.16, 0.3) | 5733.81 | 8073.65 | -0.11 (-0.24, 0.04) |
| Southern Latin America | 15-19 years | 114.91 | 153.21 | 0.05 (-0.19, 0.31) | 5105.75 | 7681.94 | -0.1 (-0.21, 0) |
| Southern Latin America | <20 years | 121.32 | 159.01 | 0.05 (-0.12, 0.25) | 23503.45 | 31023.15 | -0.13 (-0.23, -0.01) |
| Southern Latin America | Age-standardized | 151.40 | 159.18 | - | - | - | - |
| Southern Sub-Saharan Africa | <5 years | 244.78 | 220.36 | 0.18 (0.02, 0.35) | 17560.38 | 17692.62 | -0.14 (-0.3, 0.04) |
| Southern Sub-Saharan Africa | 5-9 years | 170.85 | 200.43 | 0.4 (0.21, 0.64) | 11634.29 | 16346.09 | -0.17 (-0.32, 0) |
| Southern Sub-Saharan Africa | 10-14 years | 157.02 | 185.03 | 0.39 (0.2, 0.62) | 10036.19 | 14582.7 | -0.04 (-0.14, 0.05) |
| Southern Sub-Saharan Africa | 15-19 years | 143.79 | 165.15 | 0.4 (0.2, 0.62) | 8386.69 | 11888.34 | 0.03 (-0.08, 0.15) |
| Southern Sub-Saharan Africa | <20 years | 181.69 | 193.54 | 0.31 (0.16, 0.5) | 47617.56 | 60509.76 | 0.05 (-0.06, 0.17) |
| Southern Sub-Saharan Africa | Age-standardized | 146.02 | 193.38 | - | - | - | - |
| Tropical Latin America | <5 years | 121.9 | 207.64 | 0.61 (0.4, 0.83) | 21400.56 | 35730.55 | -0.03 (-0.15, 0.08) |
| Tropical Latin America | 5-9 years | 100.76 | 204.57 | 0.81 (0.53, 1.07) | 18750.37 | 34180.54 | -0.03 (-0.18, 0.12) |
| Tropical Latin America | 10-14 years | 96.55 | 198.05 | 0.83 (0.55, 1.07) | 17167.37 | 32237.41 | -0.02 (-0.15, 0.13) |
| Tropical Latin America | 15-19 years | 90.69 | 187.05 | 0.85 (0.6, 1.11) | 14228.19 | 30666.5 | 0 (-0.14, 0.14) |
| Tropical Latin America | <20 years | 102.75 | 199.46 | 0.76 (0.51, 0.99) | 71546.5 | 132814.99 | 0.03 (-0.07, 0.14) |
| Tropical Latin America | Age-standardized | 113.21 | 199.58 | - | - | - | - |
| Western Europe | <5 years | 127.43 | 122.43 | -0.01 (-0.12, 0.09) | 29257.26 | 25989.97 | -0.4 (-0.49, -0.29) |
| Western Europe | 5-9 years | 125.8 | 121.22 | -0.02 (-0.13, 0.12) | 29587.52 | 27841.22 | 0.52 (0.35, 0.72) |
| Western Europe | 10-14 years | 123.59 | 117.61 | -0.01 (-0.13, 0.13) | 30368.44 | 28132.53 | 0.58 (0.41, 0.8) |
| Western Europe | 15-19 years | 119.55 | 115.74 | 0.01 (-0.11, 0.12) | 32701.17 | 27306.74 | 0.63 (0.46, 0.84) |
| Western Europe | <20 years | 123.89 | 119.15 | -0.01 (-0.1, 0.1) | 121914.39 | 109270.46 | -0.32 (-0.42, -0.21) |
| Western Europe | Age-standardized | 120.31 | 119.39 | - | - | - | - |
| Western Sub-Saharan Africa | <5 years | 106.01 | 183.01 | 0.17 (0.05, 0.29) | 37816.68 | 146332.91 | -0.01 (-0.14, 0.13) |
| Western Sub-Saharan Africa | 5-9 years | 43.22 | 121.47 | 0.52 (0.35, 0.72) | 12347.61 | 86912.16 | 0.01 (-0.13, 0.17) |
| Western Sub-Saharan Africa | 10-14 years | 39.74 | 115.09 | 0.58 (0.41, 0.8) | 9366.98 | 72798.97 | 0.02 (-0.13, 0.17) |
| Western Sub-Saharan Africa | 15-19 years | 36.85 | 105.08 | 0.63 (0.46, 0.84) | 7193.93 | 56546.77 | 0.05 (-0.1, 0.2) |
| Western Sub-Saharan Africa | <20 years | 62.16 | 135 | 0.34 (0.21, 0.48) | 66725.19 | 362590.81 | 0.07 (-0.06, 0.21) |
| Western Sub-Saharan Africa | Age-standardized | 94.04 | 131.90 | - | - | - | - |
