# Supplementary material for: Burden of low birth weight and short gestation from 1990–2021 and projection to 2050: assessment against 2030 malnutrition reduction targets
Source: Front Pediatr. 2025 Jun 24;13:1545857. doi: 10.3389/fped.2025.1545857 (PMC12234553; doi:10.3389/fped.2025.1545857)
Supplement: Supplementary file 2 [file Table2.docx]

**Table 3: Short gestation and low birth weight age-standardized DALYs, mortalities and YLDs in 204 countries**

|  |  | 1990 | 2021 |
| --- | --- | --- | --- |
|  |  | Rate | Rate |
| **DALYs (Disability-Adjusted Life Years)** | |  |  |
| Afghanistan | Age-standardized | 18757.31 (16081.83, 22749.71) | 6552.07 (5136.84, 7897.68) |
| Albania | Age-standardized | 4383.33 (3805.23, 5132.68) | 2243.65 (1710.95, 2810.96) |
| Algeria | Age-standardized | 8646.70 (7417.41, 10152.95) | 3033.99 (2214.06, 3933.10) |
| American Samoa | Age-standardized | 2927.88 (2524.94, 3357.69) | 1639.82 (1255.52, 2100.89) |
| Andorra | Age-standardized | 1067.04 (868.19, 1313.48) | 211.33 (135.08, 288.39) |
| Angola | Age-standardized | 20850.71 (17595.34, 24265.38) | 7297.43 (6047.77, 8666.05) |
| Antigua and Barbuda | Age-standardized | 2901.26 (2399.94, 3487.84) | 1869.85 (1578.71, 2167.78) |
| Argentina | Age-standardized | 5294.35 (5004.89, 5593.13) | 1711.15 (1312.24, 2175.86) |
| Armenia | Age-standardized | 6398.34 (5625.68, 7256.64) | 1774.32 (1450.77, 2181.86) |
| Australia | Age-standardized | 1670.00 (1544.42, 1813.76) | 671.44 (544.81, 819.50) |
| Austria | Age-standardized | 1545.20 (1419.78, 1684.49) | 675.36 (558.07, 798.64) |
| Azerbaijan | Age-standardized | 10050.37 (8725.78, 11464.22) | 5046.44 (4102.10, 6093.89) |
| Bahamas | Age-standardized | 4551.71 (3747.59, 5489.92) | 1875.62 (1455.80, 2373.20) |
| Bahrain | Age-standardized | 4320.57 (3864.94, 4904.77) | 904.85 (735.86, 1092.09) |
| Bangladesh | Age-standardized | 23174.61 (20600.90, 26335.85) | 7513.83 (5660.49, 9620.45) |
| Barbados | Age-standardized | 4422.99 (3716.35, 5185.96) | 2568.22 (1827.61, 3554.39) |
| Belarus | Age-standardized | 2272.33 (1890.79, 2735.74) | 492.15 (376.13, 628.16) |
| Belgium | Age-standardized | 1478.63 (1360.14, 1614.89) | 660.95 (524.13, 813.50) |
| Belize | Age-standardized | 7702.09 (6858.11, 8685.16) | 3198.06 (2625.76, 3856.23) |
| Benin | Age-standardized | 19496.80 (17822.66, 21304.62) | 11817.75 (9969.97, 13790.61) |
| Bermuda | Age-standardized | 2035.53 (1691.63, 2451.77) | 655.84 (452.12, 858.90) |
| Bhutan | Age-standardized | 20090.19 (17308.80, 22851.27) | 5985.94 (4560.81, 7574.39) |
| Bolivia (Plurinational State of) | Age-standardized | 9556.72 (8226.05, 11270.34) | 3139.71 (2483.92, 3813.11) |
| Bosnia and Herzegovina | Age-standardized | 4085.35 (3563.26, 4651.45) | 1211.14 (959.44, 1497.05) |
| Botswana | Age-standardized | 10348.46 (8830.42, 12202.00) | 7514.20 (5776.23, 9621.99) |
| Brazil | Age-standardized | 7732.34 (6904.65, 8524.48) | 2372.79 (1896.76, 2942.76) |
| Brunei Darussalam | Age-standardized | 1843.44 (1559.70, 2159.00) | 1776.94 (1409.58, 2280.29) |
| Bulgaria | Age-standardized | 1991.89 (1813.60, 2175.43) | 1046.53 (855.37, 1249.18) |
| Burkina Faso | Age-standardized | 18606.76 (16794.61, 20532.47) | 10408.96 (8616.57, 12470.04) |
| Burundi | Age-standardized | 15528.47 (13415.91, 17935.40) | 8239.51 (6531.46, 10233.68) |
| Cabo Verde | Age-standardized | 7013.84 (6116.42, 7898.43) | 2820.00 (2079.51, 3749.16) |
| Cambodia | Age-standardized | 15220.20 (12877.20, 18000.71) | 5185.98 (4225.96, 6305.84) |
| Cameroon | Age-standardized | 14116.48 (12273.86, 15929.80) | 8121.39 (6760.25, 9911.59) |
| Canada | Age-standardized | 1370.48 (1256.36, 1486.43) | 916.27 (751.52, 1116.10) |
| Central African Republic | Age-standardized | 20878.46 (18251.47, 23686.97) | 14032.91 (11412.60, 16999.44) |
| Chad | Age-standardized | 20018.43 (17713.67, 22434.41) | 13793.20 (11148.07, 16835.21) |
| Chile | Age-standardized | 2586.84 (2404.51, 2774.58) | 1072.18 (902.10, 1258.66) |
| China | Age-standardized | 5105.81 (4423.71, 5829.69) | 697.61 (586.61, 828.71) |
| Colombia | Age-standardized | 5976.82 (5251.76, 6744.18) | 1777.99 (1251.30, 2543.61) |
| Comoros | Age-standardized | 20981.87 (18341.03, 23909.98) | 10156.97 (8254.96, 12251.02) |
| Congo | Age-standardized | 10029.38 (8493.28, 11533.15) | 5592.42 (4392.08, 6898.97) |
| Cook Islands | Age-standardized | 3292.61 (2773.61, 3891.53) | 770.24 (594.48, 950.41) |
| Costa Rica | Age-standardized | 2926.97 (2657.22, 3207.92) | 1395.97 (1129.71, 1717.67) |
| Croatia | Age-standardized | 1973.98 (1776.09, 2201.89) | 693.88 (533.93, 880.50) |
| Cuba | Age-standardized | 2260.63 (2103.22, 2435.05) | 650.05 (552.40, 756.17) |
| Cyprus | Age-standardized | 3002.58 (2530.75, 3621.28) | 449.95 (361.30, 559.16) |
| Czechia | Age-standardized | 2251.32 (2097.94, 2419.09) | 510.30 (403.67, 635.11) |
| Cote d'Ivoire | Age-standardized | 19388.38 (17157.48, 21584.09) | 11666.51 (9778.32, 13927.95) |
| Democratic People's Republic of Korea | Age-standardized | 4503.83 (3537.56, 5679.71) | 1501.67 (1070.11, 2008.70) |
| Democratic Republic of the Congo | Age-standardized | 12331.03 (10723.05, 14155.88) | 6677.53 (5142.89, 8354.47) |
| Denmark | Age-standardized | 1348.56 (1206.95, 1512.58) | 769.70 (627.21, 927.38) |
| Djibouti | Age-standardized | 11451.86 (9952.59, 13061.17) | 5475.96 (4403.42, 6706.44) |
| Dominica | Age-standardized | 4041.89 (3370.53, 4834.53) | 5111.51 (3632.08, 7077.37) |
| Dominican Republic | Age-standardized | 11325.60 (9916.90, 12794.63) | 6281.43 (5036.83, 7725.54) |
| Ecuador | Age-standardized | 5785.16 (5027.94, 6684.77) | 2093.28 (1636.68, 2634.83) |
| Egypt | Age-standardized | 11317.93 (9676.29, 13230.44) | 1664.18 (1282.66, 2118.10) |
| El Salvador | Age-standardized | 6684.72 (5668.25, 7770.14) | 1544.31 (1131.70, 2099.52) |
| Equatorial Guinea | Age-standardized | 16031.14 (13492.57, 18708.58) | 5931.58 (4280.68, 7991.90) |
| Eritrea | Age-standardized | 13307.40 (10738.72, 16234.50) | 6895.15 (5400.29, 8757.03) |
| Estonia | Age-standardized | 2164.28 (1955.60, 2391.02) | 340.11 (275.04, 411.05) |
| Eswatini | Age-standardized | 8748.40 (7524.04, 10049.50) | 5231.28 (4038.93, 6669.32) |
| Ethiopia | Age-standardized | 23129.69 (20894.15, 25400.26) | 9864.24 (7951.79, 12132.30) |
| Fiji | Age-standardized | 3526.60 (2849.15, 4301.25) | 3048.51 (2288.68, 4049.90) |
| Finland | Age-standardized | 1144.52 (1046.15, 1243.96) | 386.09 (314.10, 462.13) |
| France | Age-standardized | 1065.11 (989.93, 1141.74) | 708.39 (566.40, 861.18) |
| Gabon | Age-standardized | 10398.05 (8925.57, 12022.92) | 5517.01 (4113.71, 7345.82) |
| Gambia | Age-standardized | 20739.48 (18191.44, 23260.53) | 9511.73 (7564.85, 11986.23) |
| Georgia | Age-standardized | 6140.93 (5281.38, 7128.56) | 1732.78 (1359.04, 2182.58) |
| Germany | Age-standardized | 1407.50 (1293.36, 1539.31) | 787.29 (653.29, 923.00) |
| Ghana | Age-standardized | 15111.14 (13452.63, 16809.72) | 6989.08 (5336.42, 9096.33) |
| Greece | Age-standardized | 1796.55 (1673.54, 1931.53) | 846.45 (691.64, 1018.86) |
| Greenland | Age-standardized | 6799.80 (5832.47, 7920.96) | 1724.39 (1375.71, 2070.22) |
| Grenada | Age-standardized | 4656.71 (3830.27, 5649.07) | 2817.14 (2256.42, 3475.48) |
| Guam | Age-standardized | 2586.41 (2255.68, 2935.37) | 2452.28 (1932.41, 3092.73) |
| Guatemala | Age-standardized | 12012.32 (10888.76, 13247.22) | 2944.20 (2236.31, 3806.18) |
| Guinea | Age-standardized | 22643.93 (19913.01, 25688.68) | 10596.71 (8667.32, 13028.80) |
| Guinea-Bissau | Age-standardized | 23301.28 (20102.55, 27037.88) | 10896.14 (8791.51, 13283.86) |
| Guyana | Age-standardized | 14079.98 (12224.65, 15998.08) | 5513.40 (4007.16, 7261.88) |
| Haiti | Age-standardized | 15048.42 (12958.82, 17073.95) | 9279.26 (7409.12, 11284.37) |
| Honduras | Age-standardized | 6413.75 (5498.55, 7430.51) | 2618.07 (1977.96, 3312.18) |
| Hungary | Age-standardized | 3909.71 (3659.99, 4150.01) | 771.55 (597.35, 962.95) |
| Iceland | Age-standardized | 1234.82 (1072.38, 1414.91) | 391.05 (311.04, 487.27) |
| India | Age-standardized | 18357.52 (16410.21, 20332.95) | 8478.75 (6804.85, 10460.18) |
| Indonesia | Age-standardized | 8805.23 (7824.51, 9807.57) | 3838.79 (3080.93, 4693.67) |
| Iran (Islamic Republic of) | Age-standardized | 9133.38 (7828.34, 10793.90) | 874.50 (690.70, 1077.48) |
| Iraq | Age-standardized | 12125.74 (10634.01, 13797.17) | 3954.67 (3088.17, 5029.25) |
| Ireland | Age-standardized | 1215.98 (1096.77, 1346.37) | 558.37 (455.00, 677.02) |
| Israel | Age-standardized | 2107.54 (1959.40, 2267.11) | 376.49 (300.21, 463.51) |
| Italy | Age-standardized | 1946.41 (1848.26, 2036.30) | 502.85 (400.75, 607.96) |
| Jamaica | Age-standardized | 6962.85 (5816.29, 8173.84) | 4231.41 (3137.11, 5649.73) |
| Japan | Age-standardized | 680.69 (631.61, 733.39) | 262.66 (217.18, 310.25) |
| Jordan | Age-standardized | 6792.19 (5856.12, 7910.68) | 2218.57 (1789.65, 2685.17) |
| Kazakhstan | Age-standardized | 4061.70 (3524.67, 4684.63) | 1644.38 (1364.91, 1997.62) |
| Kenya | Age-standardized | 9462.81 (8249.94, 10633.65) | 5690.68 (4615.98, 7001.67) |
| Kiribati | Age-standardized | 10729.18 (8619.44, 13124.52) | 5914.79 (4737.32, 7442.99) |
| Kuwait | Age-standardized | 1952.35 (1676.11, 2231.78) | 1217.83 (989.03, 1472.83) |
| Kyrgyzstan | Age-standardized | 6734.13 (5615.81, 7790.51) | 2787.36 (2330.73, 3242.34) |
| Lao People's Democratic Republic | Age-standardized | 18590.64 (15267.38, 22736.76) | 6626.52 (5094.05, 8213.63) |
| Latvia | Age-standardized | 1830.18 (1686.10, 1989.69) | 529.95 (438.39, 628.55) |
| Lebanon | Age-standardized | 6746.84 (5491.70, 8150.15) | 1493.79 (1063.67, 2070.01) |
| Lesotho | Age-standardized | 14394.08 (12588.56, 16498.84) | 10650.65 (8427.28, 12913.44) |
| Liberia | Age-standardized | 26067.19 (22752.36, 29705.34) | 9703.58 (7516.69, 12517.63) |
| Libya | Age-standardized | 5835.57 (4609.78, 7240.47) | 2769.53 (2153.55, 3532.34) |
| Lithuania | Age-standardized | 1815.87 (1647.91, 2014.39) | 474.68 (397.08, 559.42) |
| Luxembourg | Age-standardized | 1391.08 (1240.25, 1562.89) | 494.92 (399.95, 608.64) |
| Madagascar | Age-standardized | 14183.76 (12612.16, 15852.33) | 7926.93 (6321.94, 9734.06) |
| Malawi | Age-standardized | 18214.25 (16312.55, 20055.99) | 7395.88 (6105.09, 8828.53) |
| Malaysia | Age-standardized | 2975.95 (2660.54, 3395.20) | 1222.10 (1009.76, 1457.42) |
| Maldives | Age-standardized | 10739.34 (9129.85, 12483.52) | 2426.64 (1964.56, 2974.67) |
| Mali | Age-standardized | 31654.57 (28197.68, 35321.35) | 18052.50 (15169.31, 21104.68) |
| Malta | Age-standardized | 2319.86 (2074.89, 2586.65) | 1076.31 (857.77, 1340.89) |
| Marshall Islands | Age-standardized | 4901.80 (4235.25, 5590.50) | 3436.13 (2676.46, 4366.51) |
| Mauritania | Age-standardized | 17558.23 (15257.87, 20199.99) | 7719.39 (6464.61, 9301.30) |
| Mauritius | Age-standardized | 5343.49 (4847.91, 5916.37) | 3018.77 (2480.63, 3513.34) |
| Mexico | Age-standardized | 6076.39 (5503.79, 6702.64) | 2204.41 (1774.31, 2680.78) |
| Micronesia (Federated States of) | Age-standardized | 5621.54 (4558.76, 6821.41) | 2556.67 (1999.56, 3160.33) |
| Monaco | Age-standardized | 1171.09 (883.81, 1489.00) | 450.15 (371.53, 534.91) |
| Mongolia | Age-standardized | 6795.92 (5747.47, 8100.68) | 2337.06 (1906.17, 2900.75) |
| Montenegro | Age-standardized | 3412.78 (2959.29, 3866.21) | 686.53 (545.81, 835.27) |
| Morocco | Age-standardized | 12537.02 (10872.36, 14400.34) | 3006.00 (2382.85, 3648.57) |
| Mozambique | Age-standardized | 21363.31 (18118.14, 24816.95) | 9389.62 (7140.41, 12181.16) |
| Myanmar | Age-standardized | 13200.24 (11140.94, 15675.38) | 5665.13 (4559.53, 6988.30) |
| Namibia | Age-standardized | 10992.53 (9680.46, 12458.76) | 5733.11 (4497.57, 7251.94) |
| Nauru | Age-standardized | 4441.98 (3663.70, 5327.26) | 3777.28 (2785.18, 5217.12) |
| Nepal | Age-standardized | 23036.48 (19747.56, 26016.39) | 7427.76 (5648.28, 9654.60) |
| Netherlands | Age-standardized | 1367.50 (1255.59, 1485.30) | 785.33 (672.12, 901.13) |
| New Zealand | Age-standardized | 1534.66 (1395.88, 1685.02) | 872.16 (735.51, 1000.15) |
| Nicaragua | Age-standardized | 7583.52 (6507.65, 8589.13) | 2544.17 (1862.45, 3331.53) |
| Niger | Age-standardized | 21740.71 (18880.18, 24821.04) | 10908.34 (9126.76, 13133.03) |
| Nigeria | Age-standardized | 21552.06 (19802.05, 23595.48) | 14209.35 (11705.26, 17018.22) |
| Niue | Age-standardized | 3216.35 (2631.77, 3861.29) | 6594.98 (6053.20, 7192.36) |
| North Macedonia | Age-standardized | 5800.53 (5019.99, 6577.60) | 1212.81 (1013.79, 1420.72) |
| Northern Mariana Islands | Age-standardized | 2217.36 (1753.34, 2760.42) | 1273.10 (1006.59, 1559.50) |
| Norway | Age-standardized | 1496.18 (1357.06, 1632.99) | 411.64 (344.69, 485.34) |
| Oman | Age-standardized | 5480.09 (4379.59, 6649.68) | 1402.30 (1147.10, 1645.46) |
| Pakistan | Age-standardized | 19621.12 (17772.06, 21759.44) | 14312.18 (11897.40, 17249.28) |
| Palau | Age-standardized | 5587.91 (4365.30, 7137.46) | 3010.22 (2465.19, 3696.95) |
| Palestine | Age-standardized | 7690.23 (6672.10, 8851.31) | 2026.06 (1574.22, 2605.50) |
| Panama | Age-standardized | 3851.04 (3287.34, 4436.72) | 1901.35 (1456.50, 2445.12) |
| Papua New Guinea | Age-standardized | 8463.42 (7199.00, 9828.99) | 6548.12 (5291.31, 7849.02) |
| Paraguay | Age-standardized | 5760.28 (4806.14, 6773.40) | 2491.90 (1792.14, 3362.91) |
| Peru | Age-standardized | 8674.32 (7543.92, 10043.52) | 2045.46 (1405.49, 2650.23) |
| Philippines | Age-standardized | 6943.08 (5882.90, 8014.59) | 3833.17 (3075.07, 4732.58) |
| Poland | Age-standardized | 3135.91 (2936.14, 3332.65) | 725.03 (572.90, 904.31) |
| Portugal | Age-standardized | 2188.42 (2031.48, 2346.61) | 573.00 (471.51, 676.10) |
| Puerto Rico | Age-standardized | 4193.71 (3879.29, 4538.41) | 1304.90 (1073.95, 1577.85) |
| Qatar | Age-standardized | 3373.96 (2720.56, 4162.95) | 799.90 (618.84, 1007.39) |
| Republic of Korea | Age-standardized | 1818.86 (1518.58, 2173.54) | 397.48 (301.07, 503.81) |
| Republic of Moldova | Age-standardized | 3507.73 (3032.89, 4033.71) | 1409.74 (1037.98, 1880.42) |
| Romania | Age-standardized | 2555.10 (2293.56, 2840.84) | 1111.00 (973.79, 1261.78) |
| Russian Federation | Age-standardized | 2719.34 (2565.39, 2866.88) | 783.89 (687.36, 883.14) |
| Rwanda | Age-standardized | 15967.77 (13784.67, 18213.26) | 5611.40 (4354.04, 7063.95) |
| Saint Kitts and Nevis | Age-standardized | 6357.34 (5672.64, 7110.56) | 3363.98 (2643.74, 4315.40) |
| Saint Lucia | Age-standardized | 5561.63 (4653.74, 6666.92) | 4426.21 (3203.36, 5992.73) |
| Saint Vincent and the Grenadines | Age-standardized | 5860.02 (4744.57, 7100.34) | 2698.12 (2014.38, 3563.49) |
| Samoa | Age-standardized | 4274.67 (3350.01, 5338.15) | 2175.36 (1668.99, 2847.38) |
| San Marino | Age-standardized | 1453.77 (1173.24, 1764.54) | 307.47 (205.66, 430.85) |
| Sao Tome and Principe | Age-standardized | 9253.67 (8092.40, 10533.87) | 3026.77 (2181.18, 3959.13) |
| Saudi Arabia | Age-standardized | 7111.57 (5533.94, 8825.51) | 709.59 (523.42, 898.25) |
| Senegal | Age-standardized | 16305.73 (14424.51, 18464.85) | 8070.96 (6572.83, 9778.50) |
| Serbia | Age-standardized | 5766.10 (5076.72, 6659.73) | 1010.20 (855.62, 1173.68) |
| Seychelles | Age-standardized | 3441.93 (3026.97, 3918.69) | 2632.52 (2088.31, 3301.58) |
| Sierra Leone | Age-standardized | 30906.58 (27367.54, 34689.54) | 14506.95 (11370.03, 17753.76) |
| Singapore | Age-standardized | 1300.71 (1163.25, 1453.37) | 333.99 (262.14, 420.33) |
| Slovakia | Age-standardized | 2655.02 (2435.21, 2903.32) | 924.43 (751.37, 1115.46) |
| Slovenia | Age-standardized | 1324.63 (1171.28, 1491.01) | 354.18 (288.33, 428.32) |
| Solomon Islands | Age-standardized | 5518.07 (4351.89, 6899.18) | 2762.21 (2109.67, 3560.49) |
| Somalia | Age-standardized | 18155.35 (14871.68, 21203.20) | 12277.17 (9803.61, 14964.30) |
| South Africa | Age-standardized | 9853.91 (8801.98, 10987.80) | 6678.31 (5412.32, 8177.06) |
| South Sudan | Age-standardized | 18240.28 (15309.96, 21245.10) | 16999.79 (12896.49, 22013.49) |
| Spain | Age-standardized | 1423.98 (1328.62, 1525.49) | 521.93 (437.63, 601.17) |
| Sri Lanka | Age-standardized | 4825.16 (4105.60, 5495.56) | 1491.24 (1147.14, 1939.07) |
| Sudan | Age-standardized | 19484.46 (16654.34, 23438.63) | 6250.94 (5040.01, 7875.66) |
| Suriname | Age-standardized | 10225.01 (8918.29, 11674.84) | 5385.97 (4120.84, 7112.89) |
| Sweden | Age-standardized | 1093.34 (984.30, 1211.97) | 471.81 (396.48, 554.77) |
| Switzerland | Age-standardized | 1205.44 (1096.20, 1326.12) | 673.98 (554.70, 808.65) |
| Syrian Arab Republic | Age-standardized | 6986.54 (5819.62, 8261.73) | 1479.95 (1140.10, 1823.15) |
| Taiwan (Province of China) | Age-standardized | 767.56 (690.13, 856.94) | 704.43 (583.26, 829.03) |
| Tajikistan | Age-standardized | 8585.55 (7683.19, 9553.80) | 5273.82 (4334.01, 6395.97) |
| Thailand | Age-standardized | 4817.66 (4013.13, 5717.25) | 1024.83 (869.69, 1208.13) |
| Timor-Leste | Age-standardized | 15101.03 (12641.72, 17435.02) | 5856.08 (4893.45, 6979.17) |
| Togo | Age-standardized | 17252.74 (15367.31, 19090.20) | 8519.61 (6898.42, 10357.26) |
| Tokelau | Age-standardized | 3381.42 (2686.97, 4180.64) | 7171.06 (6201.29, 8126.57) |
| Tonga | Age-standardized | 3108.91 (2571.87, 3737.05) | 1642.44 (1252.55, 2155.10) |
| Trinidad and Tobago | Age-standardized | 5980.27 (4993.69, 7109.30) | 3037.31 (2312.39, 3981.58) |
| Tunisia | Age-standardized | 8744.39 (7309.04, 10250.92) | 1879.26 (1469.49, 2309.88) |
| Turkey | Age-standardized | 11802.27 (10192.91, 13825.16) | 1758.87 (1425.51, 2173.41) |
| Turkmenistan | Age-standardized | 6239.79 (5369.27, 7228.02) | 3461.67 (2804.60, 4313.94) |
| Tuvalu | Age-standardized | 9108.89 (7571.12, 10965.54) | 2929.59 (2194.93, 3784.81) |
| Uganda | Age-standardized | 14930.64 (12990.35, 16829.98) | 7678.73 (6240.94, 9463.37) |
| Ukraine | Age-standardized | 2408.33 (2061.42, 2755.45) | 940.21 (765.72, 1140.70) |
| United Arab Emirates | Age-standardized | 3967.36 (3220.91, 4809.72) | 851.29 (667.13, 1081.63) |
| United Kingdom | Age-standardized | 1580.82 (1494.95, 1672.08) | 870.08 (723.98, 1006.07) |
| United Republic of Tanzania | Age-standardized | 14269.93 (12320.15, 16187.67) | 7614.63 (5847.64, 9774.75) |
| United States of America | Age-standardized | 2143.74 (2041.27, 2250.38) | 1231.74 (1079.03, 1391.44) |
| United States Virgin Islands | Age-standardized | 4312.98 (3679.30, 4992.05) | 1142.74 (878.84, 1408.80) |
| Uruguay | Age-standardized | 3862.40 (3612.29, 4132.64) | 1037.29 (809.51, 1320.65) |
| Uzbekistan | Age-standardized | 5497.76 (4796.70, 6346.70) | 3515.08 (2830.76, 4303.77) |
| Vanuatu | Age-standardized | 5393.19 (4373.51, 6532.86) | 3372.73 (2639.05, 4193.11) |
| Venezuela (Bolivarian Republic of) | Age-standardized | 5692.96 (5263.46, 6111.62) | 3950.92 (2988.00, 5086.04) |
| Viet Nam | Age-standardized | 7339.01 (6388.42, 8432.46) | 1961.01 (1557.54, 2463.24) |
| Yemen | Age-standardized | 18559.03 (15992.59, 21868.43) | 7494.76 (6080.83, 9242.19) |
| Zambia | Age-standardized | 12589.15 (11170.67, 14094.05) | 6349.53 (4785.15, 8222.03) |
| Zimbabwe | Age-standardized | 7977.75 (7017.08, 8891.43) | 8489.16 (7050.70, 10282.05) |
| **Deaths** |  |  |  |
| Afghanistan | Age-standardized | 204.87 (175.95, 248.43) | 70.32 (55.02, 84.71) |
| Albania | Age-standardized | 47.12 (41.02, 55.14) | 23.97 (18.25, 30.01) |
| Algeria | Age-standardized | 94.55 (81.20, 110.95) | 32.52 (23.66, 42.21) |
| American Samoa | Age-standardized | 29.40 (25.69, 33.39) | 15.38 (11.69, 19.77) |
| Andorra | Age-standardized | 10.65 (8.73, 13.02) | 1.20 (0.66, 1.74) |
| Angola | Age-standardized | 230.78 (194.88, 268.47) | 79.53 (65.95, 94.33) |
| Antigua and Barbuda | Age-standardized | 29.81 (24.75, 35.64) | 18.53 (15.78, 21.18) |
| Argentina | Age-standardized | 57.08 (54.31, 59.88) | 17.22 (13.22, 21.95) |
| Armenia | Age-standardized | 69.50 (61.30, 78.71) | 18.05 (14.83, 22.10) |
| Australia | Age-standardized | 16.52 (15.63, 17.51) | 5.78 (4.80, 6.94) |
| Austria | Age-standardized | 15.77 (14.75, 16.88) | 5.89 (5.02, 6.76) |
| Azerbaijan | Age-standardized | 109.94 (95.62, 125.20) | 54.60 (44.42, 65.76) |
| Bahamas | Age-standardized | 47.71 (39.44, 57.40) | 18.21 (14.12, 23.08) |
| Bahrain | Age-standardized | 46.07 (41.40, 52.02) | 7.97 (6.62, 9.48) |
| Bangladesh | Age-standardized | 250.41 (223.12, 283.73) | 77.79 (58.61, 99.82) |
| Barbados | Age-standardized | 46.70 (39.51, 54.52) | 26.37 (18.70, 36.88) |
| Belarus | Age-standardized | 24.23 (20.29, 29.03) | 4.61 (3.56, 5.85) |
| Belgium | Age-standardized | 15.52 (14.46, 16.73) | 6.23 (5.00, 7.62) |
| Belize | Age-standardized | 83.20 (74.36, 93.55) | 33.11 (27.24, 39.89) |
| Benin | Age-standardized | 215.54 (197.28, 235.29) | 129.89 (109.61, 151.61) |
| Bermuda | Age-standardized | 19.87 (16.73, 23.56) | 5.02 (3.39, 6.56) |
| Bhutan | Age-standardized | 218.93 (188.95, 248.49) | 63.79 (48.64, 80.51) |
| Bolivia (Plurinational State of) | Age-standardized | 104.70 (90.26, 123.32) | 33.68 (26.68, 40.82) |
| Bosnia and Herzegovina | Age-standardized | 43.43 (37.96, 49.13) | 11.96 (9.60, 14.64) |
| Botswana | Age-standardized | 111.67 (95.63, 131.39) | 80.19 (61.60, 102.75) |
| Brazil | Age-standardized | 84.74 (75.72, 93.26) | 24.14 (19.37, 30.06) |
| Brunei Darussalam | Age-standardized | 17.55 (15.05, 20.28) | 17.07 (13.61, 21.95) |
| Bulgaria | Age-standardized | 20.67 (19.05, 22.26) | 10.36 (8.53, 12.22) |
| Burkina Faso | Age-standardized | 205.77 (185.89, 226.95) | 114.87 (95.10, 137.60) |
| Burundi | Age-standardized | 171.25 (148.08, 197.69) | 90.17 (71.45, 112.05) |
| Cabo Verde | Age-standardized | 75.76 (66.28, 85.03) | 29.40 (21.63, 39.24) |
| Cambodia | Age-standardized | 166.08 (140.67, 196.39) | 55.80 (45.54, 67.84) |
| Cameroon | Age-standardized | 156.13 (135.88, 176.16) | 89.26 (74.37, 108.95) |
| Canada | Age-standardized | 13.65 (12.85, 14.46) | 8.49 (7.06, 10.22) |
| Central African Republic | Age-standardized | 231.11 (202.04, 262.11) | 154.91 (125.90, 187.69) |
| Chad | Age-standardized | 222.08 (196.45, 248.90) | 152.67 (123.40, 186.28) |
| Chile | Age-standardized | 27.27 (25.60, 28.99) | 10.19 (8.72, 11.84) |
| China | Age-standardized | 55.81 (48.42, 63.69) | 6.95 (5.88, 8.24) |
| Colombia | Age-standardized | 64.73 (57.11, 72.77) | 17.83 (12.45, 25.83) |
| Comoros | Age-standardized | 230.52 (201.71, 262.43) | 109.88 (89.13, 132.40) |
| Congo | Age-standardized | 109.80 (93.24, 126.11) | 59.99 (47.11, 73.83) |
| Cook Islands | Age-standardized | 33.92 (28.65, 39.84) | 6.19 (4.81, 7.50) |
| Costa Rica | Age-standardized | 30.96 (28.36, 33.67) | 14.05 (11.41, 17.20) |
| Croatia | Age-standardized | 20.79 (18.93, 22.86) | 6.77 (5.20, 8.56) |
| Cuba | Age-standardized | 23.11 (21.79, 24.53) | 5.99 (5.18, 6.82) |
| Cyprus | Age-standardized | 32.09 (27.16, 38.63) | 3.91 (3.21, 4.78) |
| Czechia | Age-standardized | 23.74 (22.39, 25.23) | 4.25 (3.40, 5.19) |
| Cote d'Ivoire | Age-standardized | 213.96 (189.41, 237.99) | 127.81 (107.31, 152.49) |
| Democratic People's Republic of Korea | Age-standardized | 48.31 (38.02, 60.89) | 15.66 (11.15, 20.91) |
| Democratic Republic of the Congo | Age-standardized | 136.19 (118.51, 156.14) | 73.10 (56.29, 91.45) |
| Denmark | Age-standardized | 13.86 (12.58, 15.33) | 7.50 (6.16, 8.93) |
| Djibouti | Age-standardized | 125.71 (109.28, 143.01) | 58.93 (47.33, 72.09) |
| Dominica | Age-standardized | 42.24 (35.39, 50.30) | 54.14 (38.25, 75.28) |
| Dominican Republic | Age-standardized | 122.77 (107.95, 138.31) | 66.67 (53.42, 81.98) |
| Ecuador | Age-standardized | 62.49 (54.46, 72.05) | 21.76 (17.11, 27.40) |
| Egypt | Age-standardized | 122.00 (104.46, 142.21) | 15.32 (11.85, 19.50) |
| El Salvador | Age-standardized | 72.18 (61.25, 83.65) | 15.30 (11.15, 20.98) |
| Equatorial Guinea | Age-standardized | 176.57 (148.69, 205.82) | 64.01 (46.13, 86.38) |
| Eritrea | Age-standardized | 145.52 (117.56, 177.52) | 74.18 (57.98, 94.31) |
| Estonia | Age-standardized | 21.94 (20.22, 23.82) | 2.32 (1.99, 2.68) |
| Eswatini | Age-standardized | 96.10 (82.73, 110.29) | 56.80 (43.90, 72.34) |
| Ethiopia | Age-standardized | 256.39 (231.62, 281.36) | 107.52 (86.62, 132.09) |
| Fiji | Age-standardized | 36.97 (29.93, 45.07) | 31.70 (23.73, 42.31) |
| Finland | Age-standardized | 11.80 (11.00, 12.61) | 3.34 (2.82, 3.91) |
| France | Age-standardized | 10.75 (10.19, 11.28) | 6.86 (5.57, 8.25) |
| Gabon | Age-standardized | 113.19 (97.20, 130.32) | 58.64 (43.64, 78.27) |
| Gambia | Age-standardized | 227.75 (199.91, 254.99) | 101.99 (80.99, 128.57) |
| Georgia | Age-standardized | 66.97 (57.73, 77.52) | 18.17 (14.26, 22.82) |
| Germany | Age-standardized | 13.23 (12.59, 13.95) | 6.43 (5.52, 7.30) |
| Ghana | Age-standardized | 166.47 (148.49, 184.93) | 76.07 (58.08, 99.03) |
| Greece | Age-standardized | 19.26 (18.09, 20.52) | 7.69 (6.37, 9.08) |
| Greenland | Age-standardized | 73.39 (63.11, 85.38) | 17.18 (13.73, 20.50) |
| Grenada | Age-standardized | 49.24 (40.66, 59.53) | 28.93 (23.27, 35.60) |
| Guam | Age-standardized | 26.48 (23.36, 29.75) | 24.97 (19.76, 31.55) |
| Guatemala | Age-standardized | 130.61 (118.68, 143.62) | 30.40 (23.14, 39.45) |
| Guinea | Age-standardized | 250.83 (220.57, 284.42) | 116.58 (95.39, 143.39) |
| Guinea-Bissau | Age-standardized | 257.61 (222.33, 298.83) | 118.97 (95.99, 145.02) |
| Guyana | Age-standardized | 152.62 (132.79, 172.85) | 57.91 (42.00, 76.61) |
| Haiti | Age-standardized | 163.17 (140.82, 184.76) | 100.75 (80.48, 122.45) |
| Honduras | Age-standardized | 68.44 (58.88, 78.97) | 26.70 (20.15, 33.83) |
| Hungary | Age-standardized | 41.59 (39.32, 43.69) | 6.70 (5.19, 8.28) |
| Iceland | Age-standardized | 12.18 (10.77, 13.72) | 2.82 (2.33, 3.41) |
| India | Age-standardized | 199.54 (178.82, 220.29) | 88.06 (70.80, 108.41) |
| Indonesia | Age-standardized | 96.96 (86.23, 107.86) | 41.28 (33.17, 50.50) |
| Iran (Islamic Republic of) | Age-standardized | 100.69 (86.39, 119.03) | 8.56 (6.77, 10.57) |
| Iraq | Age-standardized | 131.51 (115.58, 149.28) | 40.86 (31.87, 51.86) |
| Ireland | Age-standardized | 12.33 (11.26, 13.41) | 5.03 (4.13, 5.99) |
| Israel | Age-standardized | 22.12 (20.80, 23.54) | 2.97 (2.43, 3.57) |
| Italy | Age-standardized | 20.61 (19.76, 21.38) | 4.62 (3.67, 5.58) |
| Jamaica | Age-standardized | 74.01 (62.03, 86.54) | 43.86 (32.46, 58.80) |
| Japan | Age-standardized | 6.66 (6.32, 7.01) | 1.91 (1.62, 2.19) |
| Jordan | Age-standardized | 72.96 (63.21, 84.75) | 22.16 (17.92, 26.73) |
| Kazakhstan | Age-standardized | 43.58 (38.02, 50.04) | 17.10 (14.30, 20.77) |
| Kenya | Age-standardized | 103.97 (90.70, 116.64) | 61.06 (49.63, 75.09) |
| Kiribati | Age-standardized | 116.67 (93.89, 142.56) | 63.54 (50.93, 80.06) |
| Kuwait | Age-standardized | 20.24 (17.59, 22.90) | 12.08 (9.88, 14.50) |
| Kyrgyzstan | Age-standardized | 73.46 (61.41, 84.80) | 29.76 (24.92, 34.46) |
| Lao People's Democratic Republic | Age-standardized | 204.61 (168.11, 250.32) | 72.22 (55.50, 89.44) |
| Latvia | Age-standardized | 19.29 (17.94, 20.77) | 5.08 (4.29, 5.90) |
| Lebanon | Age-standardized | 72.90 (59.47, 87.87) | 15.00 (10.59, 20.97) |
| Lesotho | Age-standardized | 158.36 (138.60, 181.17) | 116.89 (92.56, 141.65) |
| Liberia | Age-standardized | 288.73 (252.18, 329.05) | 105.93 (81.99, 136.81) |
| Libya | Age-standardized | 63.43 (50.18, 78.68) | 29.64 (23.03, 37.83) |
| Lithuania | Age-standardized | 18.69 (17.20, 20.40) | 3.91 (3.40, 4.46) |
| Luxembourg | Age-standardized | 14.35 (12.97, 15.89) | 4.51 (3.68, 5.48) |
| Madagascar | Age-standardized | 155.94 (138.80, 173.93) | 85.77 (68.37, 105.17) |
| Malawi | Age-standardized | 201.06 (180.28, 221.21) | 80.33 (66.42, 95.78) |
| Malaysia | Age-standardized | 30.50 (27.63, 34.44) | 11.21 (9.29, 13.34) |
| Maldives | Age-standardized | 116.04 (98.99, 134.59) | 24.98 (20.28, 30.54) |
| Mali | Age-standardized | 350.91 (312.76, 391.46) | 199.26 (167.45, 232.87) |
| Malta | Age-standardized | 24.40 (22.05, 26.97) | 10.49 (8.45, 13.05) |
| Marshall Islands | Age-standardized | 51.64 (44.89, 58.60) | 35.87 (27.93, 45.68) |
| Mauritania | Age-standardized | 190.07 (165.55, 218.26) | 80.93 (68.06, 97.26) |
| Mauritius | Age-standardized | 56.63 (51.78, 62.27) | 29.84 (24.64, 34.49) |
| Mexico | Age-standardized | 66.54 (60.37, 73.26) | 23.20 (18.69, 28.24) |
| Micronesia (Federated States of) | Age-standardized | 59.62 (48.51, 72.17) | 26.04 (20.47, 32.07) |
| Monaco | Age-standardized | 11.76 (8.84, 14.93) | 3.73 (3.17, 4.23) |
| Mongolia | Age-standardized | 74.37 (62.94, 88.57) | 24.86 (20.33, 30.81) |
| Montenegro | Age-standardized | 36.34 (31.71, 40.97) | 5.99 (4.80, 7.14) |
| Morocco | Age-standardized | 136.76 (118.94, 156.78) | 31.39 (24.87, 37.94) |
| Mozambique | Age-standardized | 236.17 (200.34, 274.37) | 102.36 (77.72, 132.96) |
| Myanmar | Age-standardized | 144.69 (122.29, 171.75) | 61.44 (49.45, 75.79) |
| Namibia | Age-standardized | 119.42 (105.56, 134.89) | 60.94 (47.83, 77.10) |
| Nauru | Age-standardized | 46.78 (38.68, 56.19) | 39.74 (29.10, 55.26) |
| Nepal | Age-standardized | 249.79 (214.96, 280.96) | 78.94 (60.09, 102.75) |
| Netherlands | Age-standardized | 14.18 (13.20, 15.20) | 7.57 (6.58, 8.53) |
| New Zealand | Age-standardized | 15.67 (14.46, 16.92) | 8.50 (7.27, 9.65) |
| Nicaragua | Age-standardized | 81.91 (70.49, 92.57) | 26.38 (19.16, 34.62) |
| Niger | Age-standardized | 241.00 (209.36, 275.11) | 119.92 (100.38, 144.30) |
| Nigeria | Age-standardized | 239.12 (219.69, 261.71) | 156.65 (129.05, 187.64) |
| Niue | Age-standardized | 33.57 (27.55, 40.23) | 71.08 (65.65, 77.14) |
| North Macedonia | Age-standardized | 63.03 (54.74, 71.03) | 12.33 (10.44, 14.28) |
| Northern Mariana Islands | Age-standardized | 21.95 (17.36, 27.30) | 11.73 (9.38, 14.20) |
| Norway | Age-standardized | 14.87 (13.78, 16.00) | 3.27 (2.83, 3.75) |
| Oman | Age-standardized | 58.87 (47.20, 71.28) | 13.73 (11.32, 15.95) |
| Pakistan | Age-standardized | 214.47 (194.83, 237.24) | 154.38 (128.51, 186.14) |
| Palau | Age-standardized | 59.84 (46.73, 76.49) | 31.26 (25.69, 38.36) |
| Palestine | Age-standardized | 83.35 (72.50, 95.58) | 20.69 (16.07, 26.69) |
| Panama | Age-standardized | 40.97 (35.14, 47.03) | 19.45 (14.93, 25.01) |
| Papua New Guinea | Age-standardized | 91.61 (78.16, 106.24) | 70.62 (57.11, 84.57) |
| Paraguay | Age-standardized | 61.97 (51.85, 72.72) | 25.90 (18.55, 35.15) |
| Peru | Age-standardized | 94.24 (82.29, 108.97) | 21.16 (14.43, 27.46) |
| Philippines | Age-standardized | 75.51 (63.97, 87.15) | 40.37 (32.38, 49.86) |
| Poland | Age-standardized | 33.57 (31.62, 35.46) | 6.67 (5.32, 8.39) |
| Portugal | Age-standardized | 23.15 (21.67, 24.55) | 5.08 (4.28, 5.84) |
| Puerto Rico | Age-standardized | 43.79 (41.02, 46.90) | 11.96 (10.00, 14.38) |
| Qatar | Age-standardized | 34.52 (28.09, 42.48) | 6.34 (4.92, 7.93) |
| Republic of Korea | Age-standardized | 18.94 (15.86, 22.56) | 3.46 (2.62, 4.33) |
| Republic of Moldova | Age-standardized | 37.59 (32.63, 43.00) | 14.55 (10.70, 19.51) |
| Romania | Age-standardized | 26.66 (24.20, 29.28) | 10.84 (9.64, 12.08) |
| Russian Federation | Age-standardized | 29.13 (27.65, 30.45) | 7.57 (6.72, 8.39) |
| Rwanda | Age-standardized | 176.19 (152.21, 200.73) | 61.13 (47.44, 76.97) |
| Saint Kitts and Nevis | Age-standardized | 67.77 (60.77, 75.37) | 34.98 (27.44, 44.93) |
| Saint Lucia | Age-standardized | 56.57 (47.49, 67.40) | 44.55 (32.05, 60.91) |
| Saint Vincent and the Grenadines | Age-standardized | 62.72 (50.85, 75.91) | 27.52 (20.52, 36.49) |
| Samoa | Age-standardized | 45.58 (35.76, 56.96) | 22.66 (17.33, 29.74) |
| San Marino | Age-standardized | 14.98 (12.17, 18.16) | 2.20 (1.39, 3.19) |
| Sao Tome and Principe | Age-standardized | 100.86 (88.59, 114.45) | 30.95 (22.04, 40.61) |
| Saudi Arabia | Age-standardized | 77.28 (60.22, 95.72) | 6.36 (4.66, 8.06) |
| Senegal | Age-standardized | 179.63 (159.20, 203.17) | 87.26 (71.24, 105.64) |
| Serbia | Age-standardized | 62.36 (55.08, 71.84) | 9.88 (8.51, 11.25) |
| Seychelles | Age-standardized | 35.54 (31.59, 40.14) | 26.85 (21.36, 33.71) |
| Sierra Leone | Age-standardized | 342.81 (303.65, 384.54) | 160.33 (125.61, 196.30) |
| Singapore | Age-standardized | 13.10 (11.89, 14.36) | 2.30 (1.83, 2.81) |
| Slovakia | Age-standardized | 27.63 (25.69, 29.76) | 8.81 (7.23, 10.52) |
| Slovenia | Age-standardized | 13.39 (12.06, 14.79) | 2.75 (2.35, 3.22) |
| Solomon Islands | Age-standardized | 58.63 (46.23, 73.36) | 28.54 (21.64, 36.90) |
| Somalia | Age-standardized | 200.44 (164.32, 233.86) | 135.02 (107.74, 164.47) |
| South Africa | Age-standardized | 108.10 (96.69, 120.31) | 72.04 (58.50, 88.16) |
| South Sudan | Age-standardized | 201.57 (169.32, 234.76) | 187.81 (142.38, 243.25) |
| Spain | Age-standardized | 14.74 (13.99, 15.55) | 4.80 (4.11, 5.38) |
| Sri Lanka | Age-standardized | 48.94 (42.03, 55.26) | 13.22 (10.20, 17.41) |
| Sudan | Age-standardized | 214.97 (183.74, 258.53) | 68.07 (54.98, 85.87) |
| Suriname | Age-standardized | 110.68 (96.87, 126.09) | 57.19 (43.74, 75.68) |
| Sweden | Age-standardized | 10.55 (9.80, 11.37) | 3.48 (3.05, 3.92) |
| Switzerland | Age-standardized | 12.08 (11.22, 13.02) | 6.20 (5.18, 7.33) |
| Syrian Arab Republic | Age-standardized | 74.86 (62.64, 88.29) | 14.27 (11.07, 17.52) |
| Taiwan (Province of China) | Age-standardized | 7.14 (6.62, 7.70) | 6.50 (5.49, 7.51) |
| Tajikistan | Age-standardized | 93.69 (84.09, 103.86) | 57.11 (47.00, 69.24) |
| Thailand | Age-standardized | 51.51 (43.11, 60.93) | 9.57 (8.31, 11.13) |
| Timor-Leste | Age-standardized | 164.59 (137.96, 189.86) | 62.94 (52.70, 75.02) |
| Togo | Age-standardized | 190.32 (169.56, 210.44) | 92.94 (75.27, 112.98) |
| Tokelau | Age-standardized | 35.20 (27.98, 43.48) | 77.58 (67.35, 87.73) |
| Tonga | Age-standardized | 33.53 (27.83, 40.15) | 17.30 (13.20, 22.69) |
| Trinidad and Tobago | Age-standardized | 61.23 (51.68, 72.52) | 28.70 (21.80, 37.89) |
| Tunisia | Age-standardized | 95.31 (79.74, 111.48) | 19.41 (15.24, 23.81) |
| Turkey | Age-standardized | 128.61 (111.31, 150.44) | 17.66 (14.35, 21.83) |
| Turkmenistan | Age-standardized | 68.24 (58.87, 78.84) | 37.41 (30.37, 46.64) |
| Tuvalu | Age-standardized | 99.50 (82.87, 119.67) | 30.96 (23.16, 40.06) |
| Uganda | Age-standardized | 164.66 (143.28, 185.54) | 83.76 (68.07, 103.28) |
| Ukraine | Age-standardized | 25.91 (22.27, 29.48) | 9.61 (7.88, 11.62) |
| United Arab Emirates | Age-standardized | 41.48 (33.82, 50.22) | 7.08 (5.60, 9.07) |
| United Kingdom | Age-standardized | 16.49 (15.80, 17.22) | 8.60 (7.21, 9.82) |
| United Republic of Tanzania | Age-standardized | 157.16 (135.78, 177.97) | 82.74 (63.56, 106.25) |
| United States of America | Age-standardized | 21.57 (20.95, 22.15) | 11.58 (10.34, 12.84) |
| United States Virgin Islands | Age-standardized | 44.96 (38.65, 51.78) | 9.73 (7.48, 11.99) |
| Uruguay | Age-standardized | 41.49 (39.13, 44.16) | 10.03 (7.85, 12.68) |
| Uzbekistan | Age-standardized | 60.04 (52.48, 69.17) | 38.02 (30.67, 46.56) |
| Vanuatu | Age-standardized | 57.77 (46.96, 69.85) | 35.43 (27.68, 44.08) |
| Venezuela (Bolivarian Republic of) | Age-standardized | 61.52 (57.09, 65.61) | 42.35 (32.03, 54.68) |
| Viet Nam | Age-standardized | 79.39 (69.32, 90.78) | 20.20 (16.04, 25.36) |
| Yemen | Age-standardized | 202.58 (174.76, 238.50) | 80.25 (65.16, 98.94) |
| Zambia | Age-standardized | 138.63 (123.13, 155.04) | 68.74 (51.79, 89.11) |
| Zimbabwe | Age-standardized | 86.83 (76.53, 96.50) | 92.44 (76.81, 111.77) |
| **YLDs (Years Lived with Disability)** | | |  |
| Afghanistan | Age-standardized | 326.49 (224.89, 438.69) | 225.32 (160.47, 295.67) |
| Albania | Age-standardized | 145.43 (98.48, 203.20) | 86.94 (57.56, 121.03) |
| Algeria | Age-standardized | 145.79 (98.08, 202.25) | 109.07 (73.79, 151.70) |
| American Samoa | Age-standardized | 284.41 (197.93, 382.31) | 257.41 (182.62, 338.37) |
| Andorra | Age-standardized | 109.44 (72.66, 153.34) | 103.58 (68.14, 143.99) |
| Angola | Age-standardized | 98.14 (64.69, 137.45) | 144.86 (100.02, 195.34) |
| Antigua and Barbuda | Age-standardized | 219.92 (148.31, 302.28) | 202.97 (139.10, 274.05) |
| Argentina | Age-standardized | 160.22 (109.20, 219.78) | 162.20 (112.23, 221.71) |
| Armenia | Age-standardized | 146.95 (98.94, 203.13) | 150.75 (102.77, 203.74) |
| Australia | Age-standardized | 183.77 (124.52, 258.54) | 151.18 (101.99, 209.12) |
| Austria | Age-standardized | 127.21 (83.71, 178.98) | 145.75 (99.94, 202.51) |
| Azerbaijan | Age-standardized | 160.08 (108.98, 218.05) | 134.30 (91.24, 180.36) |
| Bahamas | Age-standardized | 259.55 (179.33, 351.12) | 237.69 (161.16, 319.68) |
| Bahrain | Age-standardized | 178.59 (122.09, 248.77) | 188.37 (128.19, 256.18) |
| Bangladesh | Age-standardized | 646.25 (447.20, 882.37) | 514.64 (354.13, 697.01) |
| Barbados | Age-standardized | 221.29 (150.26, 301.42) | 196.32 (132.42, 262.65) |
| Belarus | Age-standardized | 92.37 (60.41, 131.42) | 77.30 (51.58, 108.36) |
| Belgium | Age-standardized | 82.74 (53.48, 117.67) | 100.32 (67.24, 140.36) |
| Belize | Age-standardized | 218.91 (152.90, 294.73) | 219.41 (152.26, 292.32) |
| Benin | Age-standardized | 122.15 (81.48, 171.81) | 139.83 (96.79, 188.50) |
| Bermuda | Age-standardized | 248.46 (164.72, 343.88) | 203.98 (138.16, 278.98) |
| Bhutan | Age-standardized | 393.31 (260.14, 549.21) | 246.96 (168.09, 340.87) |
| Bolivia (Plurinational State of) | Age-standardized | 138.93 (95.55, 194.40) | 109.98 (73.78, 148.13) |
| Bosnia and Herzegovina | Age-standardized | 178.23 (121.30, 248.74) | 135.12 (89.81, 187.98) |
| Botswana | Age-standardized | 301.39 (202.19, 419.73) | 299.78 (207.19, 399.48) |
| Brazil | Age-standardized | 111.01 (79.88, 144.60) | 201.14 (145.78, 258.62) |
| Brunei Darussalam | Age-standardized | 265.31 (182.52, 357.07) | 241.61 (168.44, 324.65) |
| Bulgaria | Age-standardized | 132.97 (89.30, 182.98) | 114.60 (79.54, 155.48) |
| Burkina Faso | Age-standardized | 97.91 (66.53, 133.05) | 77.66 (53.50, 107.71) |
| Burundi | Age-standardized | 123.68 (82.34, 173.80) | 128.21 (86.82, 175.23) |
| Cabo Verde | Age-standardized | 202.30 (128.07, 301.25) | 175.26 (117.78, 239.85) |
| Cambodia | Age-standardized | 280.39 (197.05, 376.71) | 165.81 (116.24, 218.79) |
| Cameroon | Age-standardized | 80.52 (53.75, 114.10) | 96.91 (66.02, 132.71) |
| Canada | Age-standardized | 143.12 (95.65, 198.18) | 152.66 (104.26, 208.13) |
| Central African Republic | Age-standardized | 88.68 (58.86, 129.77) | 99.08 (67.17, 136.03) |
| Chad | Age-standardized | 61.17 (41.04, 87.60) | 72.02 (50.09, 97.84) |
| Chile | Age-standardized | 133.94 (89.24, 181.00) | 155.13 (106.18, 212.18) |
| China | Age-standardized | 87.46 (61.93, 116.20) | 72.94 (51.84, 94.23) |
| Colombia | Age-standardized | 154.26 (104.76, 211.57) | 174.03 (120.69, 235.01) |
| Comoros | Age-standardized | 244.11 (168.62, 337.43) | 272.25 (189.38, 363.56) |
| Congo | Age-standardized | 152.84 (99.93, 219.63) | 195.73 (135.00, 267.49) |
| Cook Islands | Age-standardized | 241.45 (169.96, 326.48) | 213.33 (147.89, 281.98) |
| Costa Rica | Age-standardized | 141.67 (95.72, 196.77) | 132.43 (89.24, 179.20) |
| Croatia | Age-standardized | 103.59 (68.57, 148.73) | 84.74 (56.30, 116.77) |
| Cuba | Age-standardized | 181.68 (124.35, 244.55) | 111.05 (75.37, 151.31) |
| Cyprus | Age-standardized | 116.18 (76.99, 165.45) | 97.92 (66.25, 137.90) |
| Czechia | Age-standardized | 115.82 (77.09, 165.06) | 127.73 (86.80, 177.76) |
| Cote d'Ivoire | Age-standardized | 147.70 (100.59, 203.30) | 172.91 (122.74, 230.68) |
| Democratic People's Republic of Korea | Age-standardized | 158.93 (106.99, 218.53) | 92.71 (63.32, 126.70) |
| Democratic Republic of the Congo | Age-standardized | 78.95 (50.72, 112.89) | 101.67 (67.61, 141.87) |
| Denmark | Age-standardized | 101.95 (66.83, 142.99) | 95.01 (64.67, 134.29) |
| Djibouti | Age-standardized | 143.23 (94.30, 203.63) | 175.42 (122.45, 234.52) |
| Dominica | Age-standardized | 241.59 (165.39, 330.76) | 241.05 (165.79, 319.04) |
| Dominican Republic | Age-standardized | 283.53 (197.07, 379.48) | 283.88 (199.15, 377.37) |
| Ecuador | Age-standardized | 163.88 (113.86, 228.05) | 136.35 (92.31, 184.17) |
| Egypt | Age-standardized | 343.01 (241.43, 456.87) | 286.78 (199.22, 385.71) |
| El Salvador | Age-standardized | 193.36 (132.08, 263.21) | 167.97 (115.92, 227.17) |
| Equatorial Guinea | Age-standardized | 147.08 (98.41, 205.31) | 174.60 (119.94, 238.24) |
| Eritrea | Age-standardized | 216.40 (144.52, 293.18) | 222.91 (157.21, 298.22) |
| Estonia | Age-standardized | 190.63 (128.71, 263.18) | 131.81 (90.40, 180.73) |
| Eswatini | Age-standardized | 103.29 (69.08, 144.56) | 121.91 (82.23, 168.18) |
| Ethiopia | Age-standardized | 71.16 (48.07, 99.51) | 193.71 (137.29, 255.85) |
| Fiji | Age-standardized | 201.92 (142.97, 271.18) | 197.83 (138.85, 263.97) |
| Finland | Age-standardized | 83.24 (53.03, 120.22) | 85.40 (56.18, 118.92) |
| France | Age-standardized | 98.02 (64.52, 137.32) | 90.91 (59.85, 127.73) |
| Gabon | Age-standardized | 215.71 (145.29, 301.55) | 242.13 (168.70, 326.08) |
| Gambia | Age-standardized | 279.75 (190.77, 384.06) | 346.03 (245.01, 461.46) |
| Georgia | Age-standardized | 117.14 (74.82, 168.13) | 98.55 (66.87, 135.54) |
| Germany | Age-standardized | 217.36 (145.54, 302.05) | 208.71 (141.85, 286.37) |
| Ghana | Age-standardized | 140.91 (94.06, 195.60) | 147.14 (101.18, 203.52) |
| Greece | Age-standardized | 64.19 (41.43, 91.72) | 155.13 (102.75, 214.70) |
| Greenland | Age-standardized | 202.77 (140.75, 275.95) | 180.37 (123.47, 243.19) |
| Grenada | Age-standardized | 227.70 (158.41, 306.70) | 214.41 (147.41, 287.09) |
| Guam | Age-standardized | 204.41 (138.63, 277.91) | 207.08 (141.51, 279.53) |
| Guatemala | Age-standardized | 264.48 (184.25, 351.42) | 210.21 (150.07, 277.81) |
| Guinea | Age-standardized | 85.64 (57.18, 122.12) | 111.04 (77.36, 149.49) |
| Guinea-Bissau | Age-standardized | 144.35 (97.98, 200.29) | 196.62 (138.30, 261.94) |
| Guyana | Age-standardized | 349.75 (247.82, 465.51) | 303.28 (214.44, 400.29) |
| Haiti | Age-standardized | 368.23 (259.27, 480.85) | 214.98 (154.12, 286.22) |
| Honduras | Age-standardized | 256.35 (179.43, 344.44) | 215.84 (150.38, 291.22) |
| Hungary | Age-standardized | 168.36 (114.30, 230.38) | 169.20 (115.45, 230.71) |
| Iceland | Age-standardized | 138.99 (93.34, 192.90) | 137.19 (92.14, 192.19) |
| India | Age-standardized | 404.98 (294.11, 524.18) | 555.97 (392.47, 740.54) |
| Indonesia | Age-standardized | 82.08 (58.71, 108.97) | 124.68 (89.32, 159.85) |
| Iran (Islamic Republic of) | Age-standardized | 80.57 (56.92, 109.45) | 104.57 (74.06, 136.06) |
| Iraq | Age-standardized | 301.36 (210.12, 404.09) | 279.79 (200.86, 369.10) |
| Ireland | Age-standardized | 107.42 (71.83, 152.66) | 106.24 (71.94, 146.56) |
| Israel | Age-standardized | 118.55 (80.22, 166.43) | 109.02 (71.86, 153.92) |
| Italy | Age-standardized | 92.49 (64.70, 123.81) | 87.68 (62.30, 114.47) |
| Jamaica | Age-standardized | 305.74 (212.57, 411.55) | 285.40 (197.81, 384.64) |
| Japan | Age-standardized | 81.82 (57.41, 110.39) | 91.14 (63.26, 120.26) |
| Jordan | Age-standardized | 229.92 (156.14, 313.59) | 225.40 (158.07, 303.64) |
| Kazakhstan | Age-standardized | 142.45 (98.02, 196.86) | 106.92 (71.10, 146.58) |
| Kenya | Age-standardized | 109.75 (76.18, 149.94) | 197.76 (141.42, 261.82) |
| Kiribati | Age-standardized | 244.57 (174.74, 326.64) | 203.17 (142.53, 268.34) |
| Kuwait | Age-standardized | 132.37 (89.24, 187.50) | 131.29 (89.52, 180.32) |
| Kyrgyzstan | Age-standardized | 125.62 (82.92, 174.33) | 110.19 (75.04, 151.22) |
| Lao People's Democratic Republic | Age-standardized | 187.99 (126.36, 257.87) | 130.43 (90.48, 176.37) |
| Latvia | Age-standardized | 94.74 (61.40, 136.57) | 72.66 (47.56, 102.68) |
| Lebanon | Age-standardized | 191.60 (131.50, 263.70) | 144.72 (96.59, 196.76) |
| Lesotho | Age-standardized | 146.71 (96.40, 206.77) | 134.66 (93.41, 182.95) |
| Liberia | Age-standardized | 115.81 (78.74, 160.92) | 177.35 (122.83, 236.68) |
| Libya | Age-standardized | 131.78 (87.05, 185.29) | 105.01 (71.88, 143.58) |
| Lithuania | Age-standardized | 134.90 (90.65, 190.48) | 123.27 (83.22, 167.73) |
| Luxembourg | Age-standardized | 99.90 (65.37, 141.24) | 89.40 (59.95, 124.44) |
| Madagascar | Age-standardized | 155.42 (102.99, 217.63) | 210.40 (145.13, 284.28) |
| Malawi | Age-standardized | 126.84 (85.07, 174.95) | 169.46 (116.18, 226.39) |
| Malaysia | Age-standardized | 231.94 (158.25, 312.63) | 213.76 (150.42, 281.43) |
| Maldives | Age-standardized | 301.05 (205.86, 409.50) | 179.82 (125.68, 245.92) |
| Mali | Age-standardized | 118.34 (78.51, 163.97) | 138.07 (96.12, 184.19) |
| Malta | Age-standardized | 125.20 (82.84, 174.74) | 132.39 (89.96, 181.54) |
| Marshall Islands | Age-standardized | 256.96 (181.82, 340.30) | 210.04 (148.08, 275.90) |
| Mauritania | Age-standardized | 463.89 (323.17, 618.37) | 440.32 (311.68, 590.90) |
| Mauritius | Age-standardized | 249.14 (174.43, 335.84) | 334.83 (239.13, 440.38) |
| Mexico | Age-standardized | 91.56 (64.11, 123.12) | 117.55 (84.28, 151.10) |
| Micronesia (Federated States of) | Age-standardized | 260.53 (182.45, 351.29) | 214.68 (148.37, 286.61) |
| Monaco | Age-standardized | 113.56 (75.50, 160.27) | 114.87 (77.83, 161.00) |
| Mongolia | Age-standardized | 107.89 (73.01, 150.79) | 101.15 (68.25, 138.90) |
| Montenegro | Age-standardized | 143.97 (96.46, 200.77) | 148.10 (103.57, 201.12) |
| Morocco | Age-standardized | 234.53 (161.71, 314.05) | 182.44 (122.95, 245.31) |
| Mozambique | Age-standardized | 127.52 (85.54, 176.48) | 182.95 (125.92, 243.56) |
| Myanmar | Age-standardized | 183.72 (129.58, 253.92) | 137.18 (95.99, 183.51) |
| Namibia | Age-standardized | 248.02 (166.51, 346.16) | 250.94 (174.73, 337.30) |
| Nauru | Age-standardized | 236.12 (165.88, 314.31) | 204.01 (145.08, 268.53) |
| Nepal | Age-standardized | 562.74 (372.01, 769.88) | 325.55 (221.14, 448.24) |
| Netherlands | Age-standardized | 92.07 (60.57, 129.25) | 103.94 (69.32, 144.22) |
| New Zealand | Age-standardized | 125.53 (85.72, 175.42) | 107.97 (74.02, 145.18) |
| Nicaragua | Age-standardized | 216.48 (148.15, 291.05) | 171.19 (118.57, 229.65) |
| Niger | Age-standardized | 93.89 (63.26, 134.15) | 127.13 (88.24, 171.86) |
| Nigeria | Age-standardized | 57.68 (40.41, 78.23) | 125.76 (91.12, 162.45) |
| Niue | Age-standardized | 198.11 (138.72, 262.58) | 206.17 (145.47, 270.89) |
| North Macedonia | Age-standardized | 133.46 (90.17, 188.57) | 103.78 (71.18, 143.56) |
| Northern Mariana Islands | Age-standardized | 243.41 (168.49, 325.48) | 218.07 (151.19, 296.37) |
| Norway | Age-standardized | 158.52 (109.15, 211.83) | 117.62 (81.70, 157.24) |
| Oman | Age-standardized | 184.82 (126.17, 254.02) | 167.01 (115.27, 225.42) |
| Pakistan | Age-standardized | 325.44 (228.18, 437.35) | 423.09 (298.49, 561.87) |
| Palau | Age-standardized | 208.00 (143.46, 280.26) | 199.54 (139.50, 266.37) |
| Palestine | Age-standardized | 197.65 (132.77, 275.58) | 165.67 (113.84, 221.08) |
| Panama | Age-standardized | 165.65 (112.79, 224.15) | 151.84 (104.66, 205.02) |
| Papua New Guinea | Age-standardized | 223.01 (155.58, 298.40) | 195.15 (138.51, 256.87) |
| Paraguay | Age-standardized | 185.49 (127.59, 253.69) | 161.98 (109.78, 218.50) |
| Peru | Age-standardized | 197.81 (135.60, 267.08) | 141.92 (98.25, 191.74) |
| Philippines | Age-standardized | 151.89 (111.04, 193.94) | 201.43 (147.07, 259.96) |
| Poland | Age-standardized | 116.93 (82.81, 156.58) | 124.89 (87.91, 162.55) |
| Portugal | Age-standardized | 106.01 (71.91, 146.61) | 116.50 (77.87, 162.40) |
| Puerto Rico | Age-standardized | 253.84 (175.32, 347.28) | 228.97 (160.77, 305.92) |
| Qatar | Age-standardized | 269.39 (182.62, 371.32) | 229.78 (159.14, 310.12) |
| Republic of Korea | Age-standardized | 115.72 (78.24, 159.30) | 86.71 (57.70, 119.95) |
| Republic of Moldova | Age-standardized | 125.83 (84.71, 175.26) | 100.76 (67.70, 137.14) |
| Romania | Age-standardized | 157.58 (107.76, 215.96) | 135.68 (92.71, 185.79) |
| Russian Federation | Age-standardized | 98.88 (69.01, 131.69) | 103.25 (73.46, 135.78) |
| Rwanda | Age-standardized | 121.37 (78.55, 173.63) | 113.64 (75.49, 158.89) |
| Saint Kitts and Nevis | Age-standardized | 261.11 (178.21, 356.23) | 217.12 (151.88, 287.70) |
| Saint Lucia | Age-standardized | 472.58 (331.30, 634.96) | 418.21 (293.95, 551.22) |
| Saint Vincent and the Grenadines | Age-standardized | 218.85 (151.71, 297.56) | 222.80 (152.26, 298.78) |
| Samoa | Age-standardized | 175.56 (120.83, 239.23) | 136.96 (94.60, 184.97) |
| San Marino | Age-standardized | 107.04 (72.10, 148.37) | 109.20 (72.66, 152.24) |
| Sao Tome and Principe | Age-standardized | 188.69 (127.09, 264.98) | 242.70 (169.22, 327.81) |
| Saudi Arabia | Age-standardized | 166.03 (112.10, 230.90) | 138.09 (96.70, 188.83) |
| Senegal | Age-standardized | 158.73 (106.06, 224.82) | 222.37 (154.15, 297.22) |
| Serbia | Age-standardized | 156.64 (105.69, 216.75) | 121.72 (82.02, 166.48) |
| Seychelles | Age-standardized | 244.66 (174.19, 328.47) | 217.10 (152.56, 287.73) |
| Sierra Leone | Age-standardized | 95.54 (64.42, 134.45) | 98.27 (68.99, 132.44) |
| Singapore | Age-standardized | 122.89 (81.88, 171.85) | 127.54 (86.72, 178.02) |
| Slovakia | Age-standardized | 169.94 (112.20, 238.37) | 132.17 (89.12, 182.44) |
| Slovenia | Age-standardized | 120.20 (80.63, 169.23) | 107.02 (71.74, 147.57) |
| Solomon Islands | Age-standardized | 243.35 (171.35, 327.45) | 194.12 (137.29, 258.99) |
| Somalia | Age-standardized | 123.34 (77.92, 178.91) | 130.91 (88.31, 184.84) |
| South Africa | Age-standardized | 129.07 (88.96, 174.92) | 197.81 (141.06, 264.60) |
| South Sudan | Age-standardized | 108.39 (72.70, 149.95) | 107.60 (74.06, 148.18) |
| Spain | Age-standardized | 98.24 (64.51, 137.77) | 90.18 (60.37, 127.61) |
| Sri Lanka | Age-standardized | 422.75 (297.77, 562.89) | 302.36 (211.23, 400.90) |
| Sudan | Age-standardized | 158.59 (105.67, 224.06) | 129.59 (88.04, 173.33) |
| Suriname | Age-standardized | 269.03 (181.76, 361.86) | 241.45 (170.47, 320.07) |
| Sweden | Age-standardized | 144.87 (97.36, 199.31) | 158.45 (109.42, 213.67) |
| Switzerland | Age-standardized | 119.46 (78.17, 165.44) | 116.53 (77.16, 160.54) |
| Syrian Arab Republic | Age-standardized | 252.07 (172.10, 348.45) | 196.50 (137.35, 261.62) |
| Taiwan (Province of China) | Age-standardized | 125.73 (83.72, 173.44) | 119.43 (79.52, 164.52) |
| Tajikistan | Age-standardized | 157.22 (107.82, 215.95) | 136.17 (94.84, 182.59) |
| Thailand | Age-standardized | 184.28 (123.86, 253.78) | 164.12 (112.11, 223.02) |
| Timor-Leste | Age-standardized | 298.94 (215.14, 391.69) | 194.99 (137.57, 256.97) |
| Togo | Age-standardized | 139.88 (93.08, 195.27) | 161.55 (109.77, 221.88) |
| Tokelau | Age-standardized | 215.77 (150.86, 291.92) | 195.84 (136.73, 257.90) |
| Tonga | Age-standardized | 93.92 (63.39, 129.83) | 86.97 (59.48, 120.04) |
| Trinidad and Tobago | Age-standardized | 471.89 (325.52, 628.75) | 455.13 (317.26, 606.65) |
| Tunisia | Age-standardized | 174.50 (116.72, 241.71) | 133.45 (92.14, 178.94) |
| Turkey | Age-standardized | 235.92 (162.51, 321.40) | 170.31 (118.19, 228.87) |
| Turkmenistan | Age-standardized | 101.66 (67.20, 143.06) | 96.59 (66.22, 131.33) |
| Tuvalu | Age-standardized | 164.43 (111.37, 224.84) | 145.10 (101.63, 194.28) |
| Uganda | Age-standardized | 121.08 (80.16, 171.50) | 144.21 (100.07, 196.65) |
| Ukraine | Age-standardized | 76.95 (50.47, 109.47) | 75.79 (52.10, 104.50) |
| United Arab Emirates | Age-standardized | 236.98 (163.57, 325.51) | 214.88 (149.52, 289.33) |
| United Kingdom | Age-standardized | 98.14 (69.82, 132.16) | 96.67 (68.73, 126.07) |
| United Republic of Tanzania | Age-standardized | 132.46 (87.89, 186.12) | 171.12 (119.58, 229.98) |
| United States of America | Age-standardized | 203.70 (142.68, 272.25) | 190.17 (133.85, 250.61) |
| United States Virgin Islands | Age-standardized | 268.07 (182.06, 362.30) | 267.86 (188.02, 356.64) |
| Uruguay | Age-standardized | 130.39 (86.33, 179.32) | 135.26 (90.48, 186.95) |
| Uzbekistan | Age-standardized | 96.76 (64.51, 133.00) | 94.46 (64.94, 128.35) |
| Vanuatu | Age-standardized | 197.77 (137.30, 267.67) | 185.93 (132.44, 243.62) |
| Venezuela (Bolivarian Republic of) | Age-standardized | 159.11 (103.81, 215.91) | 140.82 (94.09, 189.65) |
| Viet Nam | Age-standardized | 196.71 (137.42, 268.02) | 143.43 (97.65, 196.93) |
| Yemen | Age-standardized | 344.20 (241.14, 459.37) | 278.29 (198.48, 374.56) |
| Zambia | Age-standardized | 121.22 (81.30, 169.57) | 165.88 (112.35, 224.58) |
| Zimbabwe | Age-standardized | 166.44 (111.71, 230.96) | 173.08 (119.25, 234.58) |
