# Supplementary material for: Burden of low birth weight and short gestation from 1990–2021 and projection to 2050: assessment against 2030 malnutrition reduction targets
Source: Front Pediatr. 2025 Jun 24;13:1545857. doi: 10.3389/fped.2025.1545857 (PMC12234553; doi:10.3389/fped.2025.1545857)
Supplement: Supplementary file 3 [file Datasheet1.pdf]

Supplementary figure 1: Forecasted LBW and short gestation (A) Global, (B) High SDI, (C) Low-middle SDI, (D) Low SDI DALYs from 2022 to 20

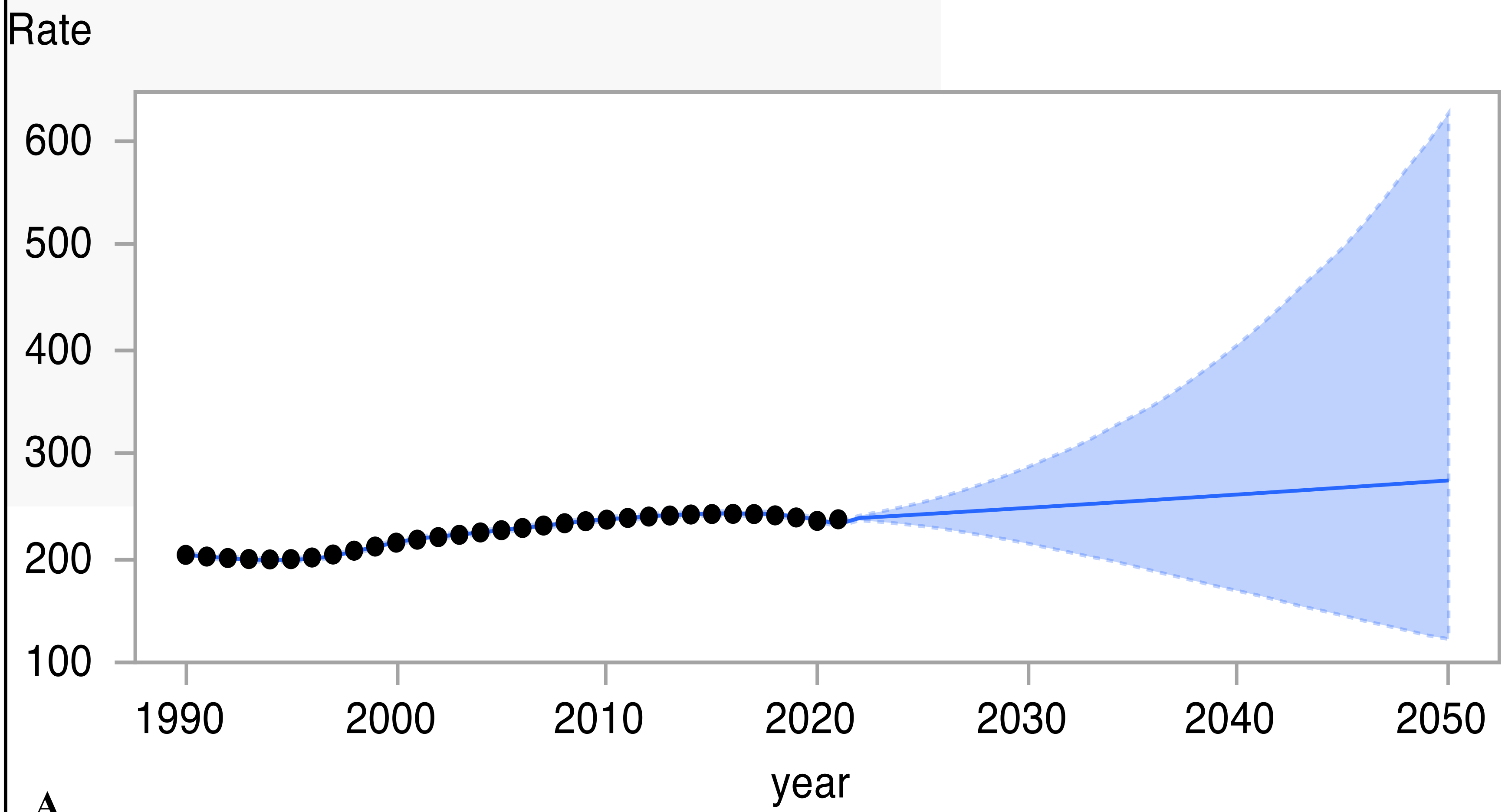

A

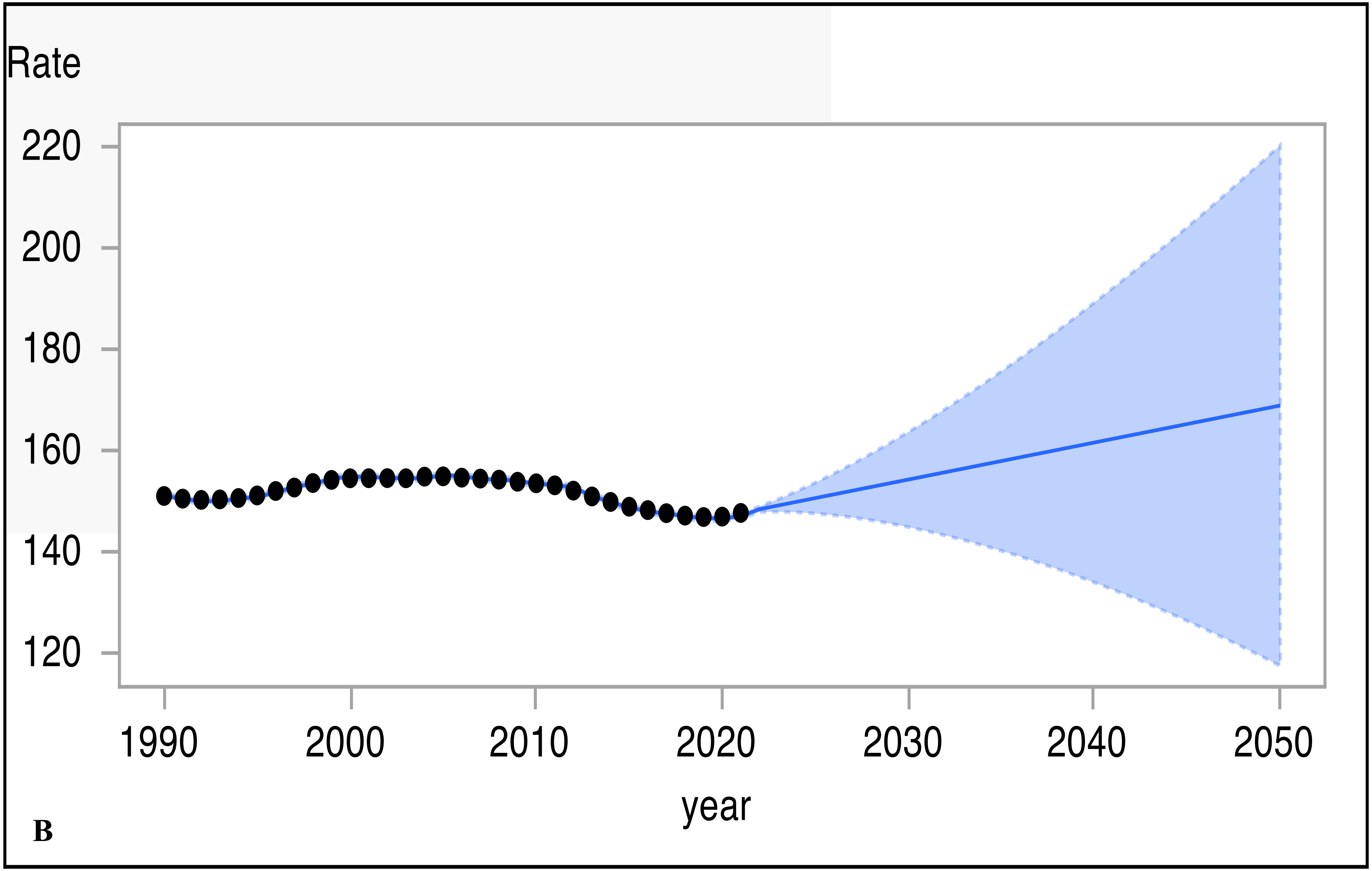

Rate
220
200
180
160
140
120
1990
2000
2010
2020
2030
2040
2050
year
B

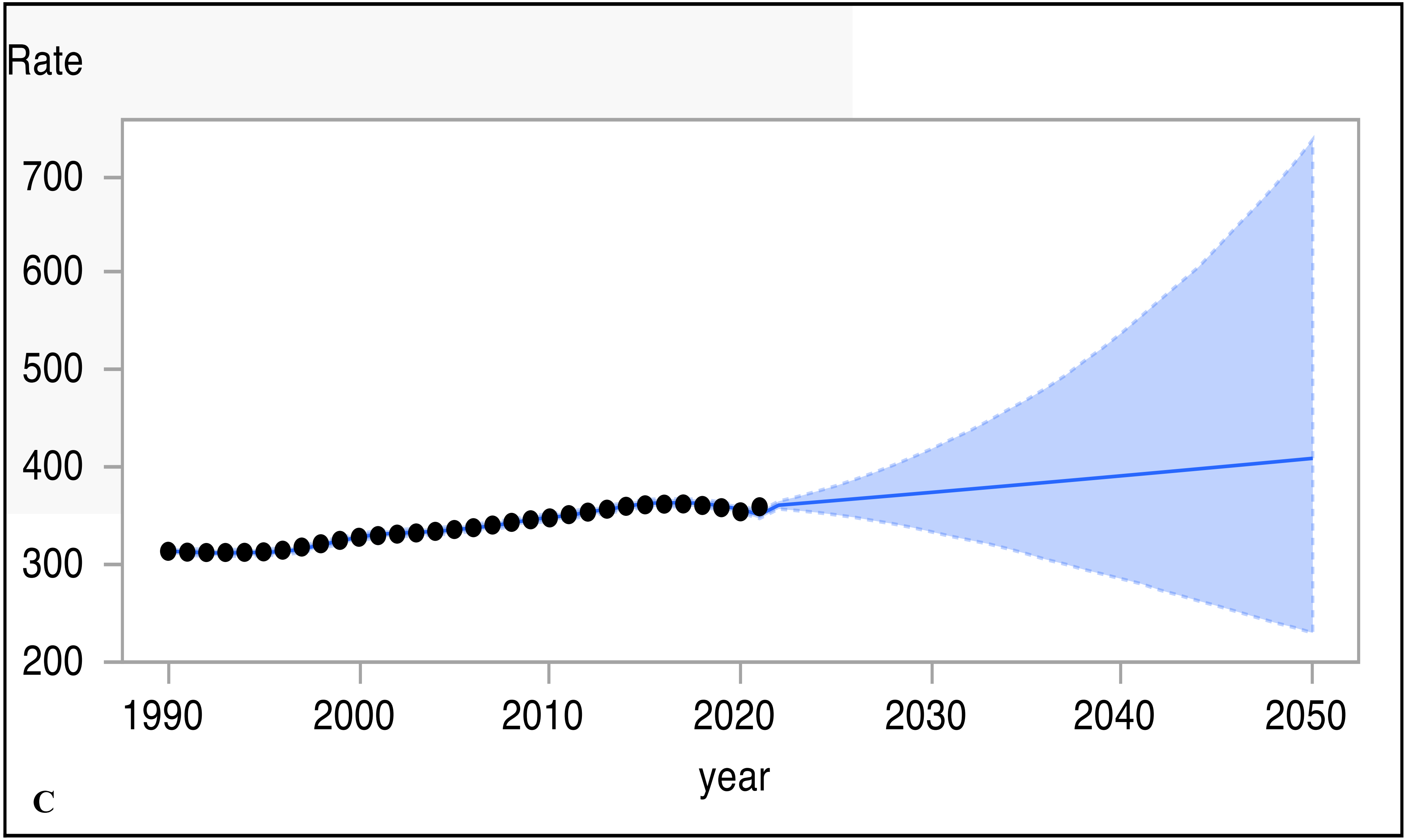

Rate
700
600
500
400
300
200
1990
2000
2010
2020
2030
2040
2050
year
C

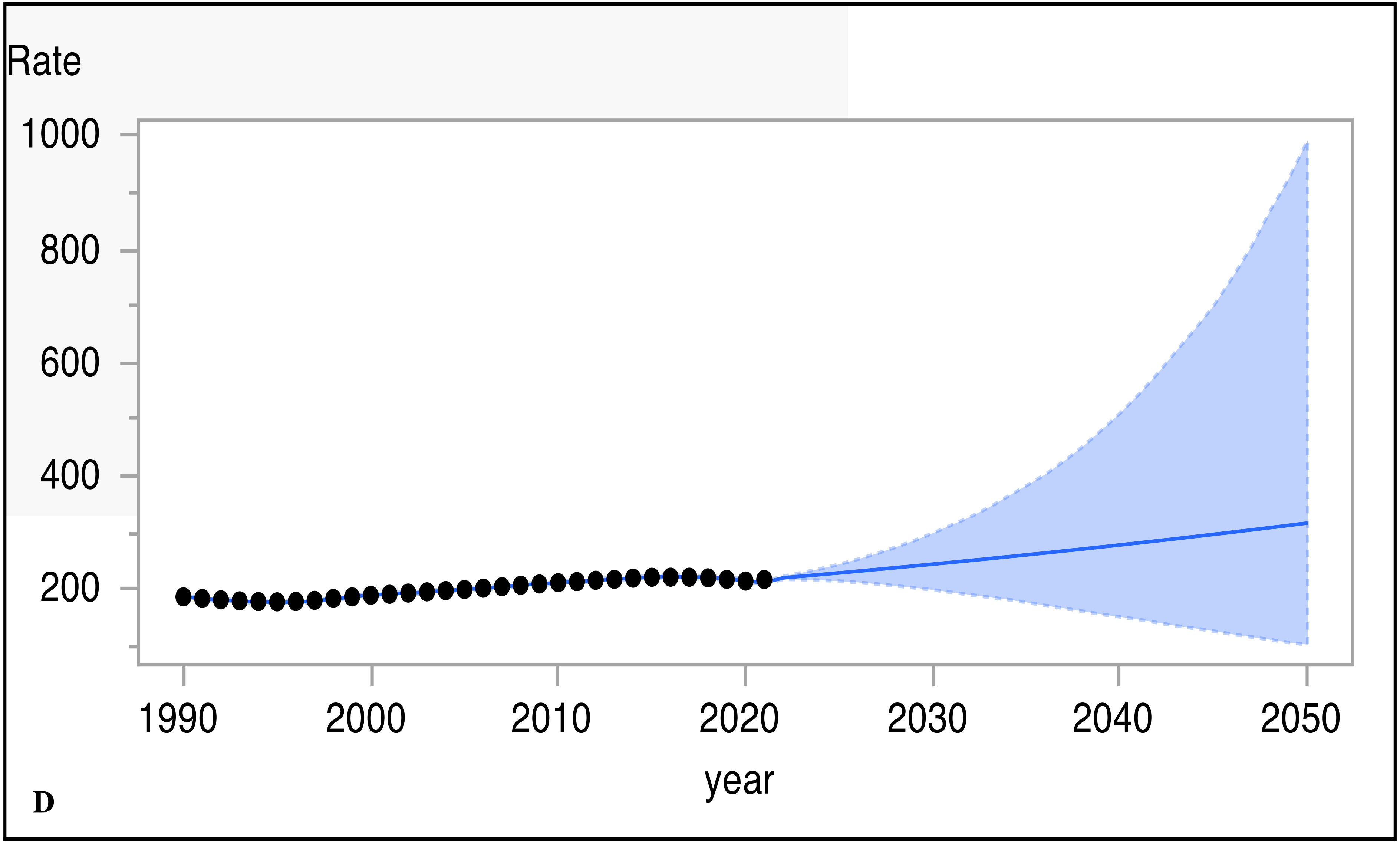
Rate
1000
800
600
400
200
1990
2000
2010
2020
2030
2040
2050
year
D
